# Bispecific antibodies and CAR T cells targeting a *TP53* mutation–associated neoantigen show discordant affinity requirements

**DOI:** 10.1172/JCI192885

**Published:** 2026-01-16

**Authors:** Sarah R. DiNapoli, Katharine M. Wright, Brian J. Mog, Alexander H. Pearlman, Tushar D. Nichakawade, Nikita Marcou, Emily Han-Chung Hsiue, Michael S. Hwang, Jacqueline Douglass, Qiang Liu, Evangeline Watson, Marco Dal Molin, Joshua D. Cohen, Maria Popoli, Suman Paul, Maximilian F. Konig, Nicolas Wyhs, P. Aitana Azurmendi, Stephanie Glavaris, Jiaxin Ge, Tolulope O. Awosika, Jin Liu, Kathleen L. Gabrielson, Sandra B. Gabelli, Drew M. Pardoll, Chetan Bettegowda, Nickolas Papadopoulos, Kenneth W. Kinzler, Shibin Zhou, Bert Vogelstein

**Affiliations:** 1 Ludwig Center, Sidney Kimmel Comprehensive Cancer Center, Johns Hopkins University School of Medicine, Baltimore, Maryland, USA.; 2 Howard Hughes Medical Institute, Chevy Chase, Maryland, USA.; 3 Lustgarten Pancreatic Cancer Research Laboratory and; 4 Bloomberg~Kimmel Institute for Cancer Immunotherapy, Sidney Kimmel Comprehensive Cancer Center, Johns Hopkins University School of Medicine, Baltimore, Maryland, USA.; 5 Department of Biophysics and Biophysical Chemistry, Johns Hopkins University School of Medicine, Baltimore, Maryland, USA.; 6 Department of Biomedical Engineering,; 7 Department of Chemical and Biomolecular Engineering, and; 8 Institute for NanoBioTechnology, Johns Hopkins University, Baltimore, Maryland, USA.; 9 Department of Oncology,; 10 Department of Surgery,; 11 Division of Rheumatology, Department of Medicine,; 12 Department of Molecular and Comparative Pathobiology,; 13 Department of Pathology,; 14 Department of Medicine, and; 15 Department of Neurosurgery, Johns Hopkins University School of Medicine, Baltimore, Maryland, USA.

**Keywords:** Immunology, Oncology, Cancer immunotherapy, Immunotherapy, T cells

## Abstract

Mutation-associated neoantigens (MANAs) are highly cancer-specific targets for immunotherapy where peptides derived from intracellular mutant proteins are presented on the cell surface via HLA molecules. T cell–engaging bispecific antibodies and CAR T cells can target MANAs to eliminate cancer cells via T cell activation. However, the low antigen density of MANAs on the cell surface can limit therapeutic efficacy. Here, we investigated whether increasing the affinity of the H2 single-chain variable fragment (scFv) targeting the p53 R175H MANA (HMTEVVRHC presented on HLA-A*02:01) improves its therapeutic effect. We identified higher-affinity H2 variants via phage biopanning and a thiocyanate elution method. Increasing bispecific antibody affinity to the low nanomolar range increased cancer cell killing and tumor control in mouse xenograft models without sacrificing antigen specificity. We next asked how increasing scFv affinity impacts CAR T cell function — a matter of debate. We appended each variant scFv to a CD28z CAR, CD3γ, or the T cell receptor. In striking contrast to the bispecific antibody results, increasing CAR affinity decreased function in each CAR format due to lower T cell activation upon interaction with target cancer cells. These results have important implications for the design of future immunotherapeutic approaches targeting low-density antigens.

## Abstract 2

Increasing binding affinity to a p53 R175H neoantigen improves anti-cancer activity for T cell-engaging bispecific antibodies and conversely decreases function for CAR T cells.

## Graphical abstract

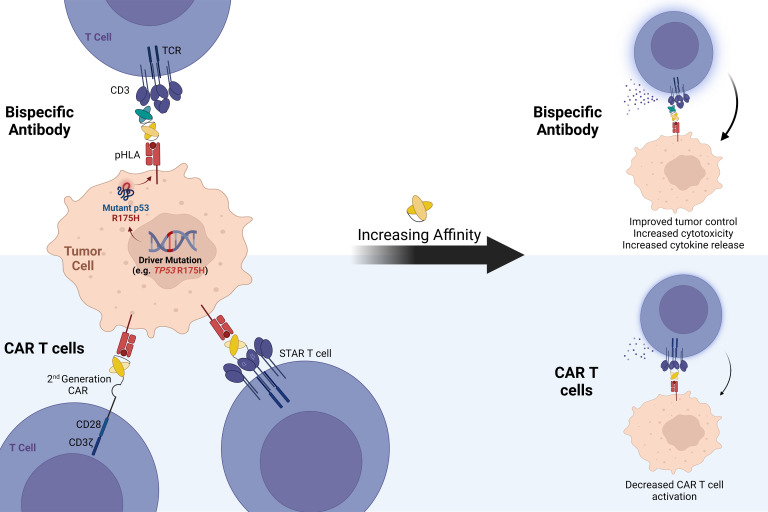

## Introduction

Immunotherapy has emerged as a promising treatment option for cancer patients with thousands of agents now in clinical development (1, 2). Mutation-associated neoantigens (MANAs) are particularly attractive targets for these agents because they are uniquely cancer specific (3–5). In contrast to tumor-associated antigens, which are also present on normal cells (e.g., MAGE-A4, CD19, and HER2), MANAs are short peptides derived from mutant intracellular proteins presented on the cell surface as peptide-HLA complexes (3). MANAs can be derived from driver mutations that occur early in oncogenesis and in intracellular proteins that are otherwise difficult to target with conventional immunotherapies or small molecules (3, 6). Because of their cancer specificity, MANAs offer a promising alternative to TAAs (7, 8). Moreover, targeting highly recurrent driver mutations such as *TP53* R175H or *KRAS* G12V has potential applications across multiple cancer types with a single therapy. For example, the *TP53* R175H mutation is one of the most common p53 mutations, present in 3%–5% of all cancers (particularly colorectal, breast, pancreatic, esophageal, ovarian, and non–small cell lung cancers) and nearly 7% of colorectal cancers (3, 9–11).

Immunotherapies can use T cell receptors (TCRs) or antibodies to recognize MANAs on the cell surface. MANA-specific TCRs can be expressed in transgenic adoptive T cell therapies (12–15) or engineered into bispecific T cell engagers (e.g., ImmTACs), where an affinity-enhanced TCR binds target cells on one end and an anti-CD3 antibody fragment (single-chain variable fragment [scFv]) binds patient T cells on the other end (16–21). Antibodies provide an alternative to TCRs for MANA recognition and can be used in bispecific T cell engager formats or as CAR T cells (4, 5, 22, 23). Antibodies generally have a much higher affinity than TCRs and can thereby improve binding to target antigens at low abundance on the cell surface (16, 18, 24–26). This increased binding is critical because MANAs are often present at less than 10 copies per cell (4, 5, 27), well below the thousands of copies per cell typically required for TAA-targeting CARs (e.g., CD19 CARs) (28–31). Now that bispecific antibodies and CAR T cells have been approved for clinical use, further work to expand target options, improve efficacy, and understand optimal characteristics — including binding affinity — may allow expanded use of these promising therapies.

We previously identified a scFv (clone H2) specific for the p53 R175H mutant peptide (HMTEVVRHC, mutation underlined) presented on HLA-A*02:01 (R175H/A2) and showed that it can be used to generate a TCR mimic bispecific antibody (MANAbody) to target cancer cells expressing p53 R175H (5). Though this R175H/A2-targeting MANAbody caused substantial regressions in p53^R175H^ mutant hematologic tumors in mice, it did not establish prolonged remissions or cure the mice. Given that antibody-based therapies that are in clinical use bind with high affinity (often < 10 nM) to their target antigens (32, 33), we hypothesized that enhancing the affinity of H2 would enhance its therapeutic efficacy. The first goal of our work was to increase the affinity of the H2 scFv. The second goal was to determine whether higher affinity improves performance of T cell–engaging bispecific antibodies in vitro and in 2 hematologic tumor models in vivo. The third goal was to employ the same scFvs, of various affinities, in CAR T cells and to determine if they perform as well as the T cell–engaging bispecific antibodies in preclinical studies.

## Results

### Selecting H2 variants with strong binding to the p53 R175H MANA.

To discover variants of the H2 scFv with improved binding affinity, we used biopanning with 4 rounds of negative and positive selection to screen a phage display library of 1,159 single–amino acid variants covering each site in the 6 complementarity determining regions (CDRs) of the H2 scFv (Supplemental Table 1 and Supplemental Methods; supplemental material available online with this article; https://doi.org/10.1172/JCI192885DS1).

We previously observed that after biopanning of a single–amino acid variant library for a KRAS-targeting MANAbody, >95% of randomly selected phage clones retained specificity for their target MANA (34). We therefore attempted to prioritize a subset of 117 randomly selected H2 variant clones after biopanning by using the previously reported crystal structure of the H2 Fab in complex with R175H/A2 (5) and identified 19 monoclonal phage clones to test for specific binding to the R175H/A2 monomer by ELISA (Supplemental Methods). Six of 19 (32%) had increased off-target binding to the WT p53 HMTEVVRRC peptide presented on HLA-A*02:01 (R175WT/A2), and 8/19 (42%) had little or no binding to the R175H/A2 monomer (Supplemental Figure 1 and Supplemental Methods). While the remaining 5/19 (26%) variants had comparable specificity to the original H2 clone by ELISA, the low yield of promising variants by structural inference led us to explore alternative methods to differentiate the H2 variants after biopanning.

We adopted a thiocyanate elution method described over 30 years ago to screen for variants with higher relative binding affinity without using structural data (35, 36). After 4 rounds of enrichment by biopanning, we applied the H2 variant phage display library to plates coated with the R175H/A2 monomer. Unbound phage were washed from the plates, and strong binders were then selected by thiocyanate elution, where plates were incubated overnight in buffer with or without 0.5 M ammonium thiocyanate (Supplemental Figure 2A). Bound phage remaining after overnight incubation were then identified by massively parallel sequencing (Supplemental Figure 2, B and C, and Supplemental Methods). Sequencing results showed that variants were enriched both through panning (Supplemental Figure 2, D and E) and with thiocyanate elution (Supplemental Figure 2, C, F, and G). Sequenced variants were ranked by their read fraction and enrichment scores (comparing variant frequency with and without thiocyanate) to select 8 scFvs for further functional testing (see Methods).

### H2 variant bispecific antibodies redirect T cells against the p53 R175H MANA.

**Figure 1:**
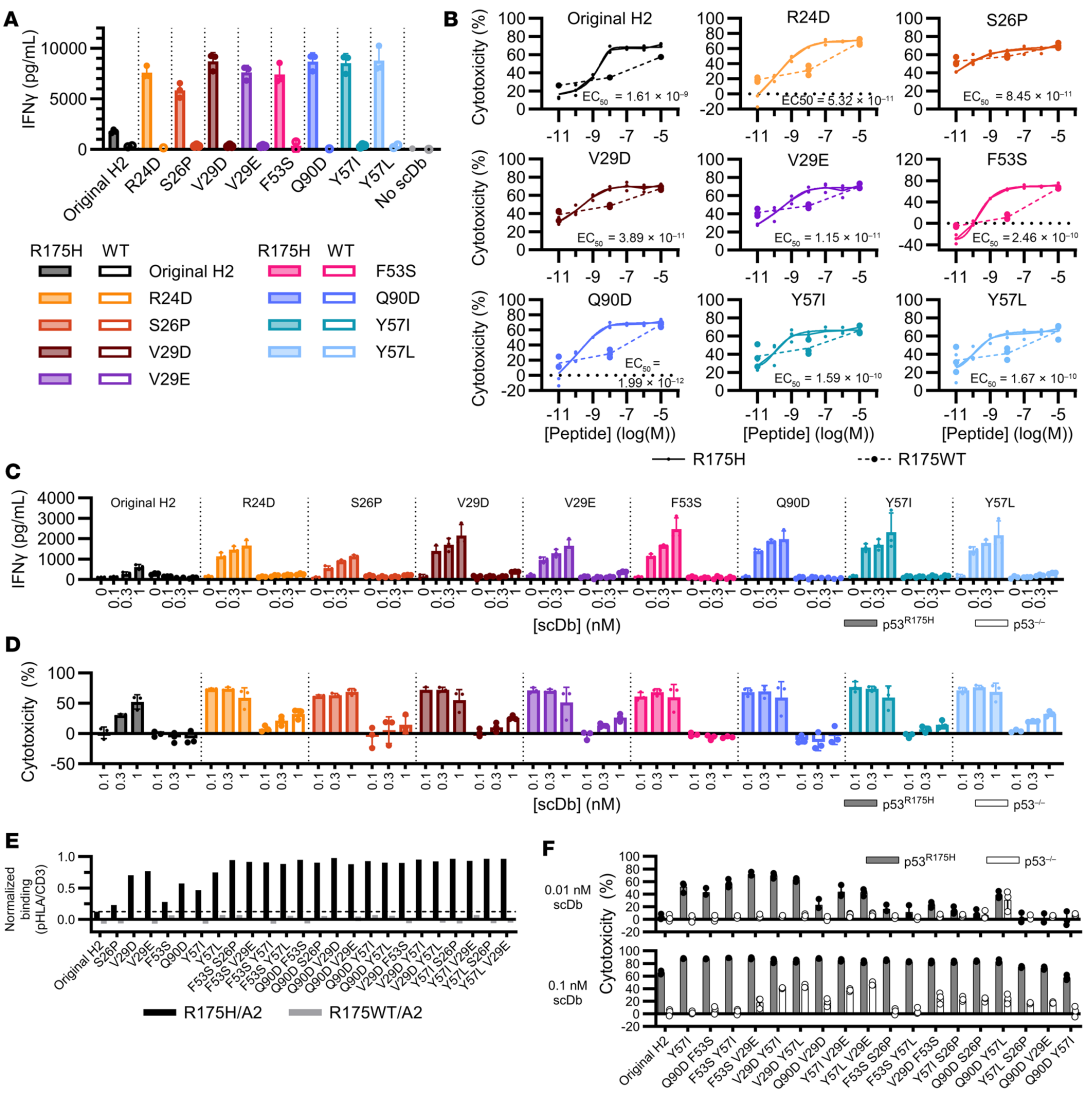
Functional testing of variants selected by thiocyanate enrichment screen. (**A** and **B**) Variants were expressed as scDbs. 5 × 10^4^ primary human T cells were cocultured overnight with 2.5 × 10^4^ T2A3 target cells pulsed with (**A**) 10 nM or (**B**) varying concentrations of the p53 R175H or R175WT 9mer peptide in the presence of 1 nM scDb. (**A**) IFN-γ production and (**B**) percent cytotoxicity compared with no scDb control by CellTiterGlo assay (*n* = 3 biological replicates, single experiment). (**C** and **D**) 5 × 10^4^ T cells were cocultured with 2.5 × 10^4^ luciferase^+^ KMS26 target cells (p53 R175H or p53 KO) and scDb for 18 hours. (**C**) IFN-γ production. (**D**) Percent cytotoxicity measured by SteadyGlo assay (*n* = 3 biological replicates, single experiment). All data are shown as the mean ± SD. (**E** and **F**) Top variants were combined into double-mutant scDbs. (**E**) scDb was applied to plates coated with CD3ε/δ heterodimer, or R175H/A2 or R175WT/A2 monomer. Relative binding was calculated by normalizing mean monomer binding to mean CD3 binding (*n* = 3 technical replicates, single experiment). (**F**) 3 × 10^4^ T cells were cocultured with 1.5 × 10^4^ KMS26 target cells in the presence of scDb for 19 hours. Percent cytotoxicity by SteadyGlo assay (*n* = 3 biological replicates per condition, single experiment).

Eight H2 variants were expressed as bispecific antibodies consisting of the p53 MANA-targeting scFv and an anti-CD3ε scFv (UCHT1) in single-chain diabody (scDb) format (4, 5). Each scDb was then tested in cocultures of primary human T cells and TAP-deficient T2A3 target cells (TAP: transporter associated with antigen processing) pulsed with the p53 R175H or WT p53 peptide. Notably, all variants selected from the thiocyanate screening stimulated substantially more IFN-γ release (Figure 1A) and cytotoxicity at low antigen density (peptide concentration) compared with the original H2 scDb (particularly evident at 10^–9^ M peptide in Figure 1B and Supplemental Table 2). The bispecific antibodies were then tested against a multiple myeloma cell line, KMS26, which has an endogenous *TP53*^R175H^ mutation and expresses approximately 2.4 copies of the R175H/A2 MANA per cell (5). Coculture with KMS26 target cells or isogenic cells in which the *TP53* gene had been inactivated via CRISPR showed that all variants selected by thiocyanate elution had increased IFN-γ release and T cell–mediated cytotoxicity compared with the original H2 scDb, particularly at low scDb concentrations (Figure 1, C and D). However, 5 of the H2 variants elicited off-target cytotoxicity against the isogenic control cells (R24D^CDRL1^, S26P^CDRL1^, V29D^CDRL1^, V29E^CDRL1^, and Y57L^CDRL2^) and were not considered further.

Variants with similar or improved potency compared with the original H2 were combined in double mutant scDbs to determine whether any pair of changes further increased bispecific antibody performance. All single and double mutants tested had increased binding to the R175H/A2 monomer compared with the original H2 scDb by ELISA, suggesting increased binding affinity (Figure 1E). Double mutants also yielded increased T cell activation in coculture with KMS26 target cells compared with H2 scDb (Figure 1F). However, 12 of the 17 variant combinations led to an increase in off-target cytotoxicity against the isogenic control cells not expressing p53 R175H (Figure 1F).

### Selected H2 variants have higher binding affinity.

**Figure 2:**
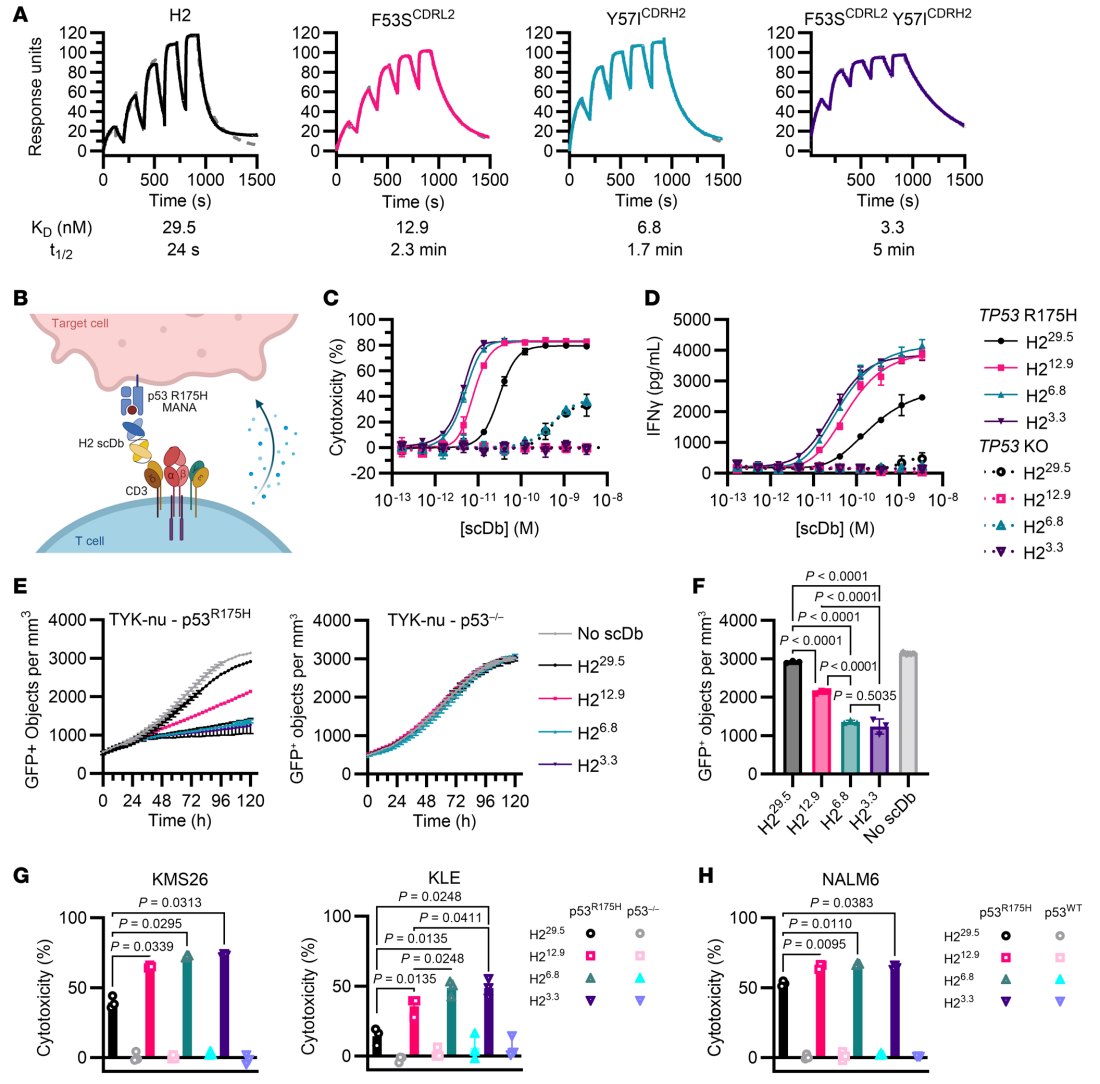
Higher-affinity bispecific antibodies have improved activity in vitro. (**A**) Single-cycle binding kinetics for each H2 variant scDb binding to the p53 R175H/HLA-A*02:01 monomer measured by SPR. Dashed line indicates curve fit. (**B**) Coculture design testing scDb cytotoxicity against isogenic p53^R175H^ and p53^–/–^ cell lines or p53^R175WT^ cell lines. (**C** and **D**) 5 × 10^4^ T cells were cocultured with 2.5 × 10^4^ KMS26 target cells in the presence of 0–3 nM scDb for 20 hours. (**C**) Percent cytotoxicity compared with no scDb was measured by SteadyGlo. (**D**) IFN-γ production (*n* = 3, representative of 3 independent experiments, curve fitting and EC_50_s determined by 5-parameter log fit). (**E**) 1 × 10^4^ GFP^+^ TYK-nu target cells were incubated with 2 × 10^4^ T cells and 0.01 nM scDb and counted by live-cell imaging (*n* = 3, representative of 2 independent experiments). (**F**) Mean GFP^+^ target cells at 120 hours (ordinary 1-way ANOVA with Tukey’s multiple comparisons). (**G** and **H**) 5 × 10^4^ T cells were cocultured with 2.5 × 10^4^ target cells and 0.01 nM scDb for 20 hours. Percent cytotoxicity compared with no scDb was measured by SteadyGlo (*n* = 3, representative of 3 independent experiments, 1-way ANOVA with Holm-Šidák multiple comparisons). All data are shown as the mean ± SD.

To determine how H2 variants differed in binding affinity, 3 scDbs with improved cytotoxicity and cytokine production were selected for affinity characterization by surface plasmon resonance (SPR): F53S^CDRL2^, Y57I^CDRH2^, and F53S^CDRL2^ Y57I^CDRH2^. The original H2 scDb had an affinity (*K_D_*) of 29.5 nM (Figure 2A and Table 1). The F53S, Y57I, and F53S Y57I variants each had higher affinities than the original H2 scDb (12.9, 6.8, and 3.3 nM, respectively), confirming that the thiocyanate elution method selected for strong binders. No variant had appreciable binding to the p53 WT/HLA-A2 monomer by SPR (Supplemental Figure 3A). All H2 bispecific antibodies had similar stability by differential scanning fluorimetry and solubility (Supplemental Figure 3, B–E, and Supplemental Methods). For convenience, these H2 scDb variants will be referred to by their affinity: H2^12.9^, H2^6.8^, and H2^3.3^. The original H2 scDb will henceforth be referred to as H2^29.5^.

### Higher-affinity H2 variants elicit stronger T cell responses against cancer cells in vitro.

We next compared the H2 scDb variants’ activity against various patient-derived cancer cell lines expressing endogenous levels of HLA-A2 and p53^R175H^ by coculture with primary human T cells (Figure 2B). All 3 higher-affinity variants had improved cytotoxicity (lower EC_50_s) against KMS26 cells compared with the original H2^29.5^ scDb (Table 2 and Figure 2, C and D). Indeed, the highest-affinity H2^3.3^ scDb had a 7 times lower EC_50_ than H2^29.5^ (4.2 vs. 31 pM). In addition, while the original H2 scDb had limited activity against TYK-nu, an ovarian cancer cell line that carries an endogenous *TP53*^R175H^ mutation and an HLA-A*02:01 allele (1.5 copies of R175H/A2 per cell) (5), the higher-affinity variants all mediated cytotoxicity against TYK-nu without off-target activity against its isogenic control cell line (CRISPR-mediated KO of the *TP53* gene) (Figure 2, E and F). H2^12.9^, H2^6.8^, and H2^3.3^ all elicited significantly improved cytotoxicity against endogenous antigen levels at very low scDb concentrations (0.01 nM) without cross-reactivity in isogenic *TP53*-KO control cell lines (Figure 2G). We also tested scDb activity against NALM6 cells edited to express p53^R175H^ via CRISPR-mediated knockin (NALM6^R175H^, 1.3 copies of R175H/A2 MANA per cell) (37) compared with the isogenic control parental NALM6 (p53^R175WT^) (Figure 2H). IFN-γ release similarly increased as affinity increased for each of the H2 variants (Supplemental Figure 4). Taken together, increasing H2 MANAbody affinity yielded increased T cell activation against cells expressing endogenous levels of the p53 MANA without an increase in off-target activity.

### Increasing bispecific antibody affinity improved tumor control in vivo.

**Figure 3:**
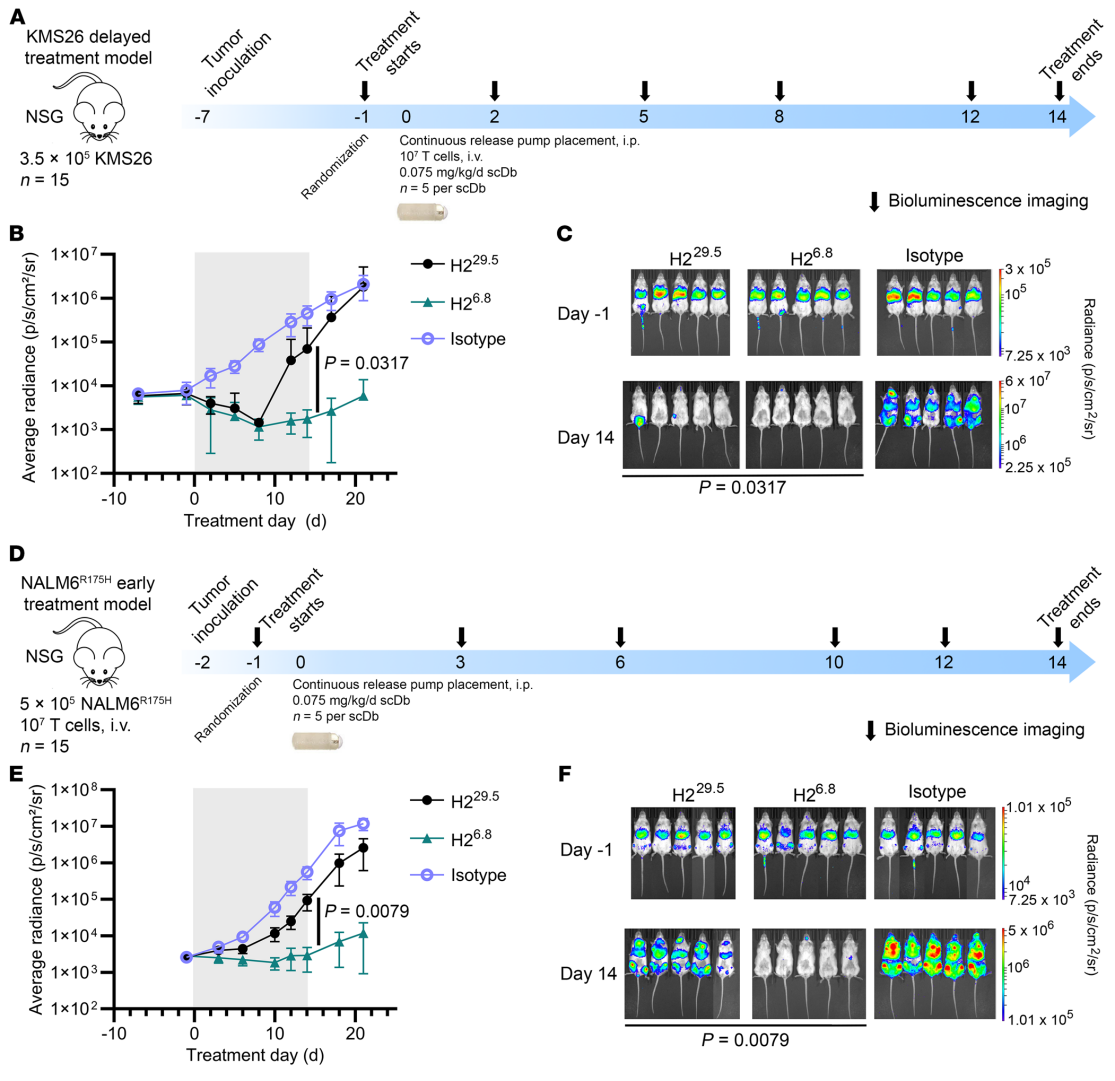
Higher-affinity H2^6.8^ has improved tumor control in vivo. (**A**) Delayed treatment model design. NSG mice were inoculated with 3.5 × 10^5^ KMS26 cells i.v. 7 days prior to treatment. On treatment day 0, mice received 10^7^ human T cells i.v. and surgically implanted micro-osmotic continuous release pumps i.p. scDb was dosed at 0.075 mg/kg/d for 14 days. Tumor burden was measured by bioluminescence imaging. (**B**) Tumor burden and (**C**) bioluminescent images at days –1 and 14 (average radiance, means compared at day 14 by Mann-Whitney test, *n* = 5 per group; data are shown as mean ± SD). (**D**) Early treatment model design. NSG mice were inoculated with 5 × 10^5^ NALM6^R175H^ cells and 10^7^ human T cells i.v. 2 days prior to treatment start. On treatment day 0, mice received surgically implanted continuous release pumps i.p. scDb was dosed at 0.075 mg/kg/d for 14 days. (**E**) Tumor burden and (**F**) bioluminescent images of mice at days –1 and 14 (average radiance, means compared at day 14 by Mann-Whitney test, *n* = 5 per group, representative of 2 experiments; data are shown as mean ± SD). Pump treatment period indicated by gray boxes.

We chose the H2^6.8^ variant for in vivo testing given its potency in the absence of any detectable off-target toxicity at low antibody concentrations (10 pM; Figure 2, G and H). We previously reported that the original H2^29.5^ MANAbody can limit KMS26 growth in a NOD.*Cg-Prkdc^scid^Il2rg^tm1Wjl^*/SzJ (NSG) mouse hematologic xenograft model (5). We used a considerably more challenging variation of this model in the current study. Specifically, we employed a delayed treatment model in which mice were treated 7 days after tumor inoculation with 3.5 × 10^5^ KMS26 cells i.v. (Figure 3A). Mice were inoculated with 10^7^ primary human T cells i.v. and treated with a low dose of bispecific antibody (0.075 mg/kg/d vs. previously reported 0.15–0.3 mg/kg/d) (5) delivered via a 14-day continuous release pump i.p. Measurement of tumor burden by bioluminescence imaging showed that mice treated with H2^6.8^ had significantly lower tumor burden at the end of treatment compared with mice treated with H2^29.5^ (day 14, *P =* 0.0317, *n* = 5 per group; Figure 3, B and C, and Supplemental Figure 5A). Moreover, tumors were undetectable in more mice at day 21 in the H2^6.8^ variant group (3/5 mice) compared with those in the H2^29.5^ group (0/5 mice) (Supplemental Figure 5A).

We additionally tested H2^6.8^ in an aggressive tumor model employing NALM6^R175H^ cells, which divide rapidly in mice. Mice were treated with a low scDb dose (0.075 mg/kg/d) via continuous release pump 2 days after receiving 5 × 10^5^ NALM6^R175H^ cells and 1 × 10^7^ human T cells i.v. (Figure 3D). In this model, the original H2^29.5^ scDb did not control tumor growth, while mice treated with the higher-affinity H2^6.8^ scDb induced tumor regression that lasted the duration of infusion (14 days). Tumor burdens on day 14 were significantly lower in the mice treated with H2^6.8^ versus the original H2^29.5^ (day 14, *P* = 0.0079, *n* = 5 per group; Figure 3, E and F, and Supplemental Figure 5B). Treatment with bispecific antibodies was well tolerated with stable body weights in all animals over the duration of treatment (Supplemental Figure 5C).

### Increasing H2 scFv affinity does not improve CAR function.

Low antigen density presents a challenge for CAR T cell therapies and is a major limitation for MANA-targeting CARs (31). While some groups have shown that increased scFv affinity increases CAR activation at low antigen density (38, 39), others have shown that higher-affinity CAR scFvs have reduced T cell proliferation and activation (40–42). In most cases, comparisons of different affinities involved unrelated scFvs, complicating conclusions about the reasons for the differences in performance. Because the H2 variants all recognize the identical, precisely defined epitope and differ by only 1 or 2 amino acids, they provide an ideal opportunity to assess the influence of affinity on CAR T cell performance.

To test the higher-affinity H2 variants in CAR T cells, we first generated second-generation CD28-CD3ζ CAR T cells (2G CAR T cells) (43) by CRISPR knockin at the *CD3G* locus in primary human T cells (Supplemental Figure 6, A and B). We chose this method of creating CAR T cells, rather than lentivirus transduction, to ensure similar expression of each construct. Edited CAR T cell frequency was analyzed by flow cytometry with no significant differences in CD4^+^ or CD8^+^ T cell frequencies or expression levels across CAR types (Supplemental Figure 6, C–F).

The 2G CAR T cells were then cocultured with TAP-deficient T2 target cells pulsed with exogenous p53 R175H 9mer peptide to test for T cell activation at different antigen densities. At 24 hours, all higher-affinity H2 2G CAR T cells had lower IFN-γ production compared with the original H2 2G CAR (Supplemental Figure 7, A and B), which was precisely the opposite of what was observed with the corresponding bispecific antibodies (Figure 2, C and D). This decreased cytokine production with increasing scFv affinity was observed at high peptide pulsing conditions (1–10 μM), while lower peptide concentrations elicited minimal 2G CAR T cell activation for all 2G CARs (Supplemental Figure 7, A and B).

### CD3γ TCR CARs improve efficacy at low antigen densities.

Second-generation CARs are known to have limited activity when antigen levels drop below 100–1,000 copies per cell (44), making targeting MANAs with traditional CARs challenging. The H2 2G CARs constructed with the original H2 MANAbody had limited activity at low p53 R175H/A2 antigen density, consistent with previous work (37). One strategy to increase CAR sensitivity is to append the scFv to the TCR complex, which may increase activity by better activating canonical immunological synapse signaling (37, 45–47). We next appended the H2 scFv to the CD3γ subunit in the TCR complex to generate a CD3γ-TRuC T cell (TRuC: T cell receptor fusion construct) (45) (Supplemental Figure 6, A, B, and G–J). We observed increased T cell activation with the CD3γ-TRuC compared with the 2G CAR (Supplemental Figure 7, C and D, compared with A and B). However, a similar trend of decreasing sensitivity with increasing scFv affinities was observed for the CD3γ-TRuC T cells (Supplemental Figure 7, C and D).

### STAR T cells mimic native TCR structure and have increased tetramer binding with higher affinity.

**Figure 4:**
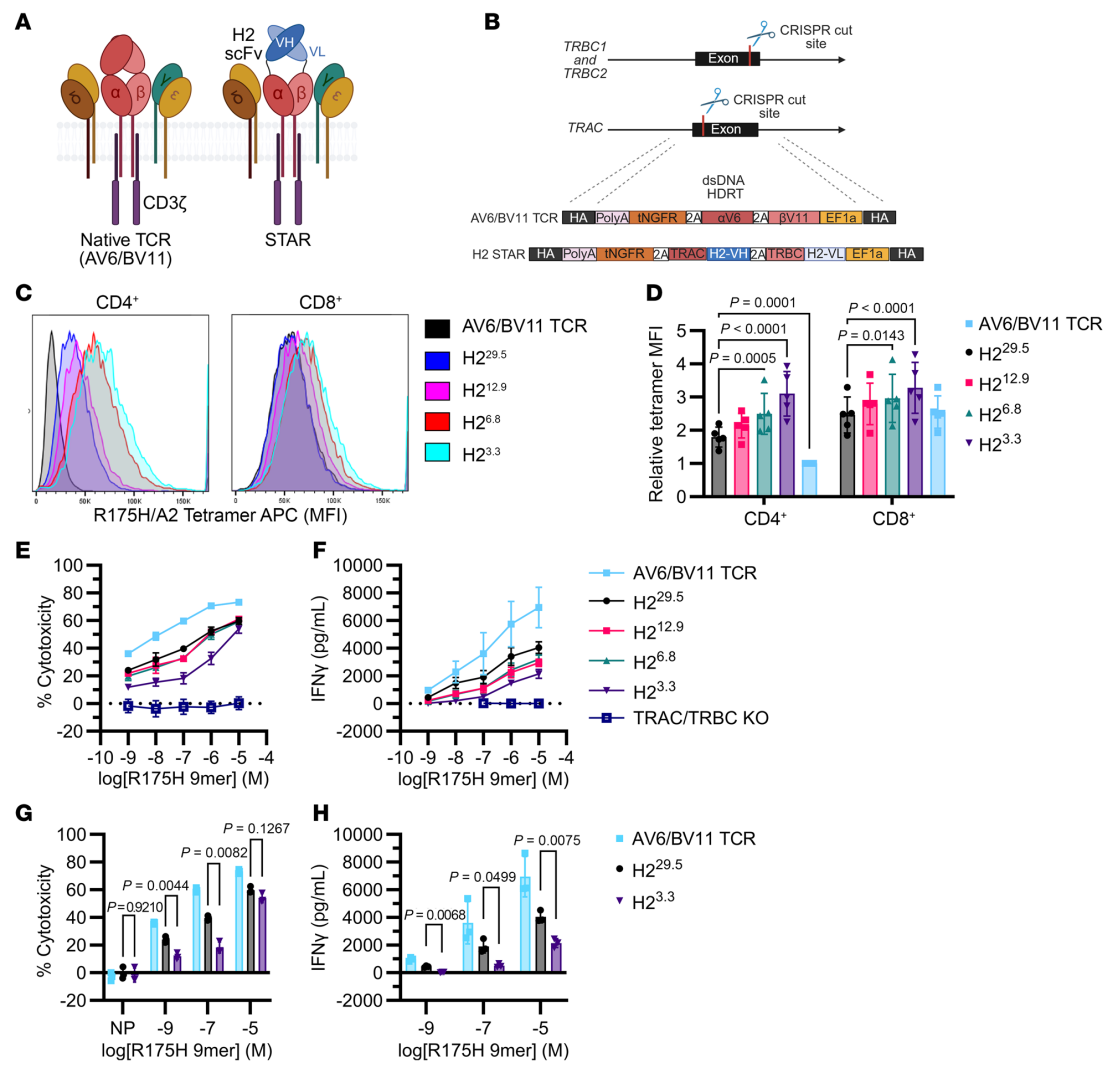
Higher-affinity STARs have increased tetramer binding but reduced antigen sensitivity. (**A**) Illustration of the patient-derived TCR AV6/BV11 compared with the H2 STAR, where the H2 variable heavy (VH) and variable light (VL) chains are linked to the TCRα constant domain (TRAC) and TCRβ constant domain (TRBC), respectively. (**B**) Transgenic T cells were generated by CRISPR knock-out at TRBC1/TRBC2 and knockin at TRAC using dsDNA homology-directed repair templates (HDRTs). HA, homology arm. (**C**) T cells expressing STARs were characterized by flow cytometry. Representative histograms of p53 R175H/HLA-A2 tetramer bound to edited (CD3^+^ tetramer) CD4^+^ and CD8^+^ T cells are shown. (**D**) Tetramer staining on CD4^+^ and CD8^+^ T cells was compared across *n* = 5 independent experiments by normalizing tetramer MFI to the tetramer MFI for CD4^+^ AV6/BV11 TCR T cells for each experiment (2-way ANOVA with Dunnett’s multiple comparisons to H2^29.5^). (**E**–**H**) T cell activation at varying antigen densities was tested in a peptide-pulsing coculture. 8 × 10^3^ edited T cells were cocultured with 4 × 10^4^ peptide-pulsed T2 cells for 24 hours. (**E**) T cell–mediated cytotoxicity was measured by CellTiterGlo viability assay. (**F**) IFN-γ production. (**G**) Cytotoxicity and (**H**) IFN-γ were compared at selected concentrations from **E** and **F** (1-way ANOVA with Dunnett’s multiple comparisons, *n* = 3 biological replicates, representative of 3 independent experiments). All data are shown as the mean ± SD.

We and others have shown that antigen sensitivity can be improved using TCR-based CAR T cells called STARs (synthetic TCR and antigen receptor T cells) (37, 46, 47). STARs consist of an engineered receptor where the CAR scFv is divided into variable heavy and light chains appended to the TCRα and TCRβ constant domains. To determine whether we could improve CAR T cell MANA targeting with higher-affinity STARs, we generated H2 STAR T cells with CRISPR editing at the *TRAC* locus to append the H2 scFv variable light chain and variable heavy chain to TCRα and TCRβ constant domains, respectively, using a modified murine *TRAC* and *TRBC* to maximize TCR assembly (Figure 4, A and B) (37, 48). We also evaluated a standard TCR (AV6/BV11) derived from a patient harboring a *TP53*^R175H^ mutant cancer (49). The only difference between this TCR and the STAR was that the variable chains of AV6/BV11 TCR were substituted for the variable chains of the H2 scFv. Both were appended to the constant regions of the modified murine TCR (Figure 4, A and B) (37).

To confirm that the variant STAR receptors were able to bind to the p53 R175H MANA, we measured p53 R175H/A2 tetramer binding by flow cytometry. Relative tetramer binding increased with increasing receptor affinity for each variant in both CD4^+^ and CD8^+^ subsets, with greater differences in tetramer binding observed in CD4^+^ T cells than in CD8^+^ T cells (Figure 4, C and D, and Supplemental Figure 8, A–F). There was no difference in receptor expression, as indicated by CD3 staining (Supplemental Figure 8, B and E). All H2 STAR variants had increased tetramer binding compared with the patient-derived TCR AV6/BV11, which is consistent with TCRs’ lower affinity compared with antibodies (44, 50). We also assessed the purity of edited STAR T cells after bead-based positive selection for T cells expressing truncated nerve growth factor receptor (tNGFR; a marker for transgene expression) and showed that edited cells did not have differences in CD4^+^ or CD8^+^ T cell frequencies (Supplemental Figure 8, C and D).

### Increasing CAR affinity does not improve STAR T cell function.

In a coculture with peptide-pulsed T2 target cells, H2 STAR T cell–mediated cytotoxicity and IFN-γ production decreased as receptor affinity increased (Figure 4, E and F). This decreased T cell activity with increasing CAR affinity was seen both at supraphysiologic antigen densities (1E-5 M peptide) and at low antigen density (1E-9 M peptide, tens of copies per cell) (16). The lower-affinity AV6/BV11 TCR T cells had the highest sensitivity and activity of all T cells tested (Figure 4, E–H).

**Figure 5:**
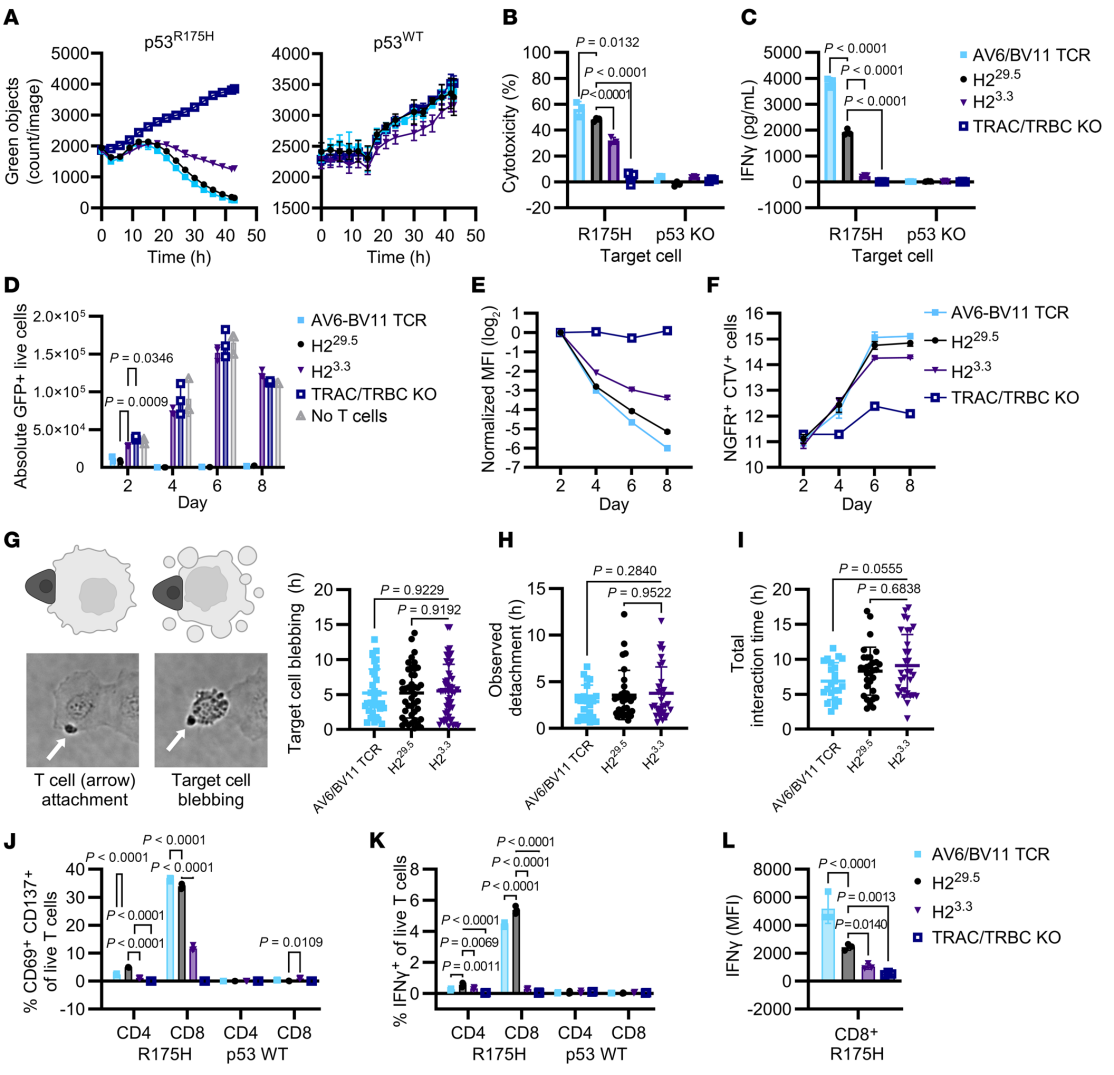
STAR activity decreases with increasing receptor affinity. (**A**) 5 × 10^4^ edited T cells were cocultured with 1 × 10^5^ GFP-expressing NALM6^R175H^ or NALM6^WT^ target cells for 2 days. (**B** and **C**) 1.2 × 10^4^ edited T cells were cocultured with 2.4 × 10^4^ KMS26 target cells for 24 hours. (**B**) Percent cytotoxicity by SteadyGlo assay compared with the TRAC/TRBC-KO condition. (**C**) IFN-γ production (*n* = 3 per condition, 2 independent experiments, 2-way ANOVA with Dunnett’s multiple comparisons). (**D**–**F**) STAR T cell multiple stimulation assay. 4 × 10^3^ edited T cells were cocultured with 1.6 × 10^4^ KMS26 target cells in replicate plates and rechallenged with 3.2 × 10^4^ target cells or assayed by flow cytometry on days 2, 4, and 6. (**D**) Live GFP^+^ target cells; (**E**) CellTrace Violet (CTV) dilution, indicative of cell division, in NGFR^+^-edited T cells relative to day 2; and (**F**) counts of NGFR^+^ T cells (*n* = 3 per condition, 3 independent experiments). (**G**–**I**) T cell killing of KLE target cells was observed by live-cell imaging (aggregate of 3 independent experiments per T cell type, Brown-Forsythe and Welch’s ANOVA with Dunnett’s T3 multiple comparisons to H2^3.3^). (**G**) Time from T cell interaction to target cell blebbing (arrows indicate T cells). (**H**) Time from target cell blebbing to T cell detachment. (**I**) Total duration of interaction. (**J**–**L**) 5 × 10^4^ edited T cells were cocultured with 1 × 10^5^ NALM6^R175H^ or NALM6^WT^ target cells for 17 hours and assayed by flow cytometry (*n* = 3 per condition, 2 independent experiments). (**J**) Percent CD69^+^ CD137^+^ of live T cells (2-way ANOVA with Dunnett’s multiple comparisons to H2^29.5^). (**K**) Percent IFN-γ^+^ of live T cells (2-way ANOVA with Dunnett’s multiple comparisons to H2^29.5^). (**L**) MFI of IFN-γ in CD8^+^ T cells (ordinary 1-way ANOVA with Šidák’s multiple-comparison test to H2^29.5^). All data are shown as the mean ± SD.

We then tested the H2 variant STAR T cells’ activity against cell lines with the endogenous *TP53*^R175H^ mutation. In cocultures with NALM6^R175H^ cells, the higher-affinity H2^3.3^ STAR T cells killed fewer target cells (Figure 5, A and B) and produced less IFN-γ (Figure 5C) than the lower-affinity H2^29.5^ STAR T cells, again the opposite of the analogous bispecific antibodies (Figure 2, C–H). We additionally employed a multiple-stimulation assay (MSA) in which STAR T cells were rechallenged with KMS26 target cells every 2 days (Supplemental Figure 9A). The H2^3.3^ STAR T cells did not reduce target cell numbers after the first rechallenge, unlike the lower-affinity H2^29.5^ STAR T cells or the AV6/BV11 TCR cells, which continued to kill target cells upon multiple challenges (Figure 5D). Similar results were observed in an MSA with NALM6 target cells (Supplemental Figure 9B).

Proliferation analysis demonstrated that fewer higher-affinity STAR T cells divided over the 8-day MSA than did the lower-affinity STAR T cells (Figure 5, E and F, and Supplemental Figure 9, D–F) despite persistence of the H2^3.3^ STAR T cells (Supplemental Figure 9C). Quantitatively, KMS26 cells stimulated only a 4.7-fold expansion in H2^3.3^ STAR T cells compared with 32.7- and 22.1-fold expansions with AV6/BV11 TCR T cells and H2^29.5^ STAR T cells, respectively (Supplemental Figure 9F). Finally, higher-affinity H2^3.3^ STAR T cells produced less IFN-γ in both MSAs (Supplemental Figure 9G).

### Investigations into the basis for the lower cytotoxicity of the higher-affinity STAR T cells.

We sought to understand the mechanistic basis for the decreased cytotoxicity observed with the higher-affinity STAR T cells. One potential explanation for this decrease was less efficient target cell killing per T cell. To test this hypothesis, we performed time-lapse live-cell imaging of the H2^3.3^ and H2^29.5^ STAR T cells in coculture with KLE target cells expressing very low levels of the p53 R175H MANA (1.3 copies/cell) (5). KLE cells were chosen for these experiments because they are adherent and therefore more suitable for live-cell imaging than nonadherent cells, such as KMS26 or NALM6 cells. Similarly to KMS26 or NALM6 cells, KLE cells were less susceptible to killing by the high-affinity H2^3.3^ STAR T cells than by the lower-affinity H2^29.5^ STAR T cells (Supplemental Figure 10, A–C). There was no difference in CAR T cell activity against isogenic control KLE p53^–/–^ cells compared with TRAC/TRBC-KO T cells (Supplemental Figure 10B). Additionally, after 6 days of coculture with KLE cells, the high-affinity H2^3.3^ STAR T cells had less expression of the late activation marker HLA-DR and less proliferation than the lower-affinity H2^29.5^ STAR T cells (Supplemental Figure 10, D–F). The relative order of the extent of cytotoxicity to target cells and cell division of T cells obtained with the various STAR T cells and the AV6/BV11 TCR were identical with all 3 target cell types (Supplemental Figure 10, A–F, Supplemental Figure 9, B–F, and Figure 5, A, B, and D–F).

We further examined the STAR T cell cytotoxic activity by manually tracking individual T cell interactions with KLE target cells with live-cell imaging. We observed T cell cytotoxic activity against KLE cells from T cell engagement with target cell–to–target cell blebbing (Figure 5G). There were no significant differences in time to target cell blebbing among H2^29.5^ STAR, H2^3.3^ STAR, and the AV6/BV11 TCR cells (Figure 5G). We also considered the possibility that the higher-affinity STAR T cells remained adhered to the target cells for longer, thereby limiting their ability to detach from one target cell and reform a synapse with another target cell. To test this possibility, we measured the interaction time between the various T cells and the target cells. We found no significant differences in time to detachment from dying cells or in total interaction time between the 3 T cell types and their target cell (Figure 5, H and I). We considered whether the higher-affinity STAR T cells themselves underwent cell death during or after target cell interactions, thereby limiting their killing potential, but we did not observe any instances of T cell apoptosis during or after a target cell interaction in the time-lapse experiments.

The data described in Figure 5, G–I, suggested that, once a T cell bound to a target cell, the resulting cytotoxic effect was similar regardless of receptor affinity. We therefore considered whether reduced activity of the high-affinity H2^3.3^ STAR T cells was due to decreased activation. To assess this hypothesis, we measured T cell expression of activation markers CD69 and CD137 after coculture with NALM6 cells for 17 hours (Figure 5J). There was indeed a striking difference in activation, with the activation of the AV6/BV11 TCR and lower-affinity H2^29.5^ STAR T cells much higher than that of the high-affinity H2^3.3^ STAR T cells (Figure 5J). These differences were particularly evident in CD8^+^ T cells but were also observed in CD4^+^ T cells (Figure 5J). Little or no activation was observed when the same T cell types were cultured with NALM6^R175WT^ cells, supporting the specificity of the CD69/CD137 marker upregulation (Figure 5J). Similarly, fewer H2^3.3^ STAR T cells upregulated CD69 and CD25 in a 48-hour coculture with NALM6 target cells (Supplemental Figure 11, A, C, and E). In contrast, in parallel cocultures with scDbs, CD69/CD25 upregulation increased with increasing scDb affinity (Supplemental Figure 11, B and D).

As an additional measure of activation, we assessed the number of cells expressing IFN-γ in the same 17-hour cocultures. The fractions of AV6/BV11 TCR and lower-affinity H2^29.5^ STAR CD8^+^ T cells that expressed IFN-γ were significantly higher than that of the high-affinity H2^3.3^ STAR CD8^+^ T cells (Figure 5K). Not only was the fraction of IFN-γ–expressing cells lower, but the amount of IFN-γ per cell was significantly decreased (Figure 5L). Finally, though increasing receptor affinity had a greater effect on CD4^+^ T cell tetramer binding compared with CD8^+^ T cells (Figure 4, C and D, and Supplemental Figure 8, E and F), CD4^+^ cells secreted much lower levels of IFN-γ than the corresponding CD8^+^ cells, regardless of the receptor types (Figure 5K).

T cell activation-induced cell death (AICD) and exhaustion are 2 potential contributors to the decreased tumor cell killing and proliferation we observed in the higher-affinity STAR T cells. To examine this, we conducted an extended MSA where T cells were challenged with Nalm6^R175H^ cells every 2 days and characterized by flow cytometry on day 14 (Supplemental Figure 12, A–C). As seen in single-stimulation experiments, fewer H2^3.3^ STAR T cells expressed early activation markers (CD69 and CD25; Supplemental Figure 12D). There was no indication of increased AICD in the higher-affinity STAR T cells. Instead, H2^3.3^ STAR T cells were less likely to have FasL expression (Supplemental Figure 12F). Across conditions, most T cells expressed Fas, which we attributed to exposure to IL-2 in the MSA and IL-2/IL-7 during T cell expansion (51). Finally, we assessed whether T cells that had been activated over the MSA had differing expression of inhibitory markers, such as Lag3, PD1, and Tim3, suggesting T cell exhaustion. We found that inhibitory marker expression corresponded with T cell activation, as expected. H2^3.3^ cells were less likely to express HLA-DR or any inhibitory markers on day 14 of the MSA (Supplemental Figure 12, H and J). Even among HLA-DR^+^ cells, the higher-affinity H2^3.3^ STAR T cells were less likely to express inhibitory markers (Supplemental Figure 12L), again suggesting that these cells were not activated to the same degree as the H2^29.5^ STAR T cells or AV6/BV11 TCR. Importantly, we did not observe signs of tonic signaling causing T cell activation, or FasL or inhibitory marker expression in the T cell–only condition (Supplemental Figure 12, E, G, I, and K).

## Discussion

Developing antibodies that recognize MANAs is challenging: the MANAbody must recognize a single amino acid difference in a 9– or 10–amino acid peptide embedded within a much larger HLA groove. It was therefore not clear that the affinity of the originally described H2 MANAbody directed against the p53 R175H/HLA-A2 complex could be improved without sacrificing specificity for the mutant peptide (24). Indeed, a standard affinity maturation approach, even when coupled with structural insights, was not successful. Fortunately, an approach described decades ago — employing thiocyanate elution — allowed us to develop higher-affinity variants. When incorporated into T cell–engaging bispecific antibody format, these high-affinity variants proved more potent in vitro and in mice. This is consistent with previous work showing that increasing bispecific antibody affinity improves potency (18, 24, 25). In this study, we used 2 hematologic models to differentiate our bispecific antibody affinity variants’ activity in vivo. How these antibodies perform against solid tumors remains of significant interest given p53 R175H’s frequency in common solid organ cancers and the additional challenges of targeting solid tumors with immunotherapies.

We expected the higher-affinity variants to lead to more potent engineered T cell therapies. This expectation was consistent with some studies, though others have shown that lower-affinity CAR T cells have improved performance (16, 38–40). It was difficult to extrapolate from these previous studies of CAR T cells employing various affinity antibodies because all were directed against cells bearing 100- to 1,000-fold higher densities of the target antigen. In fact, we have previously shown that standard CAR T cells cannot recognize cells with antigen densities as low as typically found in MANAs (37). To recognize the p53 R175H/A2 MANA, the CAR scFv had to be embedded within the endogenous TCR rather than expressed as a fusion with CD28 or other costimulatory domains (37). The hybrid TCR/antibody-based engineered T cells we developed in previous publications (37, 46, 47), described as STARs or HIT CARs (HIT: HLA-independent TCRs), enabled us to rigorously determine the relationship between affinity and function using cell-based and bispecific antibody-based therapeutic approaches.

The results of these experiments were definitive: higher affinity led to better function of the bispecific antibodies, as assessed by cytotoxicity, cytokine release, and T cell activation (Figures 1–3). Yet, higher affinity led to worse function of cell-based therapeutic approaches using the identical assays (Figures 4 and 5). Moreover, the specificity of the observed effects in both bispecific antibodies and T cells was meticulously controlled with isogenic target cells differing only in the single amino acid at codon 175 of the *TP53* gene or with *TP53* KO.

Several possible mechanisms could underlie the decreased potency of the STAR T cells bearing the higher-affinity antibodies. Differences in cell surface expression, epitope binding, or stability could potentially impact CAR function. It was therefore important to note that the variants differed by only 1 or 2 amino acids and had similar solubilities and melting temperatures (Supplemental Figure 3, B–E). We documented that the differences in potency were not due to decreased CAR expression levels on the cell surface (Figure 4C and Supplemental Figure 8, B–F). This similarity in expression across the different T cells compared in this study was expected given that they all were created through genetic knockin at the same locus rather than through methods leading to overexpression or varied levels of expression. We also demonstrated that the higher-affinity antibodies on the STAR T cells were properly folded, as evidenced by R175H/HLA-A2 tetramer binding (Figure 4C and Supplemental Figure 8, B–F).

Another possibility for the decreased potency was that higher-affinity CAR T cells had increased cell death. For example, receptor–ligand interactions could potentially result in trogocytosis — pulling target molecules from the target cell membrane to the T cell membrane — resulting in nonlethal target cell interactions and potential CAR T cell fratricide, though the limited activation seen with H2^3.3^ argues against this mechanism (42, 52). Alternatively, tonic signaling or T cell exhaustion (due to high-affinity receptor interactions or tonic signaling) could limit the H2^3.3^ T cells’ activity and lead to increased T cell death. In general, we did not observe signs of increased cell death in high-affinity CAR populations; high-affinity STARs persisted in extended cocultures and in multiple stimulation assays (Figure 5F, Supplemental Figure 9C, and Supplemental Figure 10D) and did not express markers of AICD (Supplemental Figure 12, F and G). We did not conclude that the higher-affinity STAR T cells were exhausted given that even HLA-DR^+^ H2^3.3^ STAR T cells had less Lag3, PD1, and Tim3 expression than activated H2^29.5^ or AV6/BV11 T cells.

To assess whether there was less efficient cell killing per T cell, we performed time-lapse live-cell imaging of the various STAR T cells in coculture with target cells. There were no significant differences noted in these experiments as far as the average time to target cell death (blebbing), the average interaction time between T cells and target cells, or the average time to T cell detachment from the target cells once killed (Figure 5, G–I, and Table 3). In fact, the only functional difference between the STAR T cells bearing the higher-affinity receptors was in their level of activation, as assessed by typical activation markers (Figure 5J and Supplemental Figures 10–12). These experiments support the idea that the decreased function in the higher-affinity STAR T cells is primarily related to decreased activation (or quality of activation; e.g., CD69 expression without CD25 upregulation or proliferation). Once a threshold for activation is met, we hypothesize that all the STAR T cells would then have similar abilities to kill target cells. According to this hypothesis, we expect that some T cells interacting with target cells would not form mature synapses or adhere to target cells because they weren’t sufficiently activated. We could not test this in our live-cell imaging experiments because the technique only allowed us to visualize and track relatively stable interactions between the T cells and target cells. Transient interactions, shorter than the 30-second interval between images or at the molecular level, would not have been observed in these experiments.

Future work to evaluate why higher-affinity receptors yield less frequent activation are planned, including testing how high-affinity STARs compare to TCRs as mechanoreceptors with single-molecule biophysical techniques (optical tweezers) (53, 54). Further transcriptomic or phosphoproteomic comparison of activated STAR T cells may yield additional insight into how affinity affects T cell function; however, these studies primarily address the cells’ response to activation rather than how receptor differences determine T cell activation.

These results have potentially important implications for immunotherapies based on MANAs or other low-antigen-density targets. Increasing affinity of the antibody for the target had predictable and expected effects on function when the immunotherapeutic agent was a soluble T cell–engaging bispecific antibody. This is consistent with decades of research showing that high-affinity drugs perform better than their lower-affinity counterparts, provided specificity is maintained (16, 18, 24–26). But for cellular immunotherapies, conclusions about relationship between performance and affinity have varied, as observed in our study and in previous studies of CAR T cells directed against cells expressing high levels of antigens (39–41, 55, 56). CAR T cells thereby present a Goldilocks situation, wherein the optimum antibody affinity to be used cannot be predicted — a much more challenging case for clinical development than encountered with soluble antibodies. Perhaps a better understanding of the complex interactions between CAR T cells and their targets, and the mechanisms through which activation and cytotoxicity is achieved (24, 55, 57–60), will address this quandary.

## Methods

### Sex as a biological variable.

Our in vivo studies exclusively examined female mice because they are less aggressive than male mice and therefore easier to work with. It is unknown whether the findings are relevant for male mice.

### Cell lines.

SW620, RPMI-6666, HCT116, LS123, KLE, and T2 cells were obtained from the American Type Culture Collection (ATCC). TYK-nu and KMS26 were purchased from the Japanese Collection of Research Bioresources Cell Bank. HEK293FT cells were purchased from Invitrogen (Thermo Fisher Scientific). T2A3 cells (HLA-A3–expressing T2 cells) were provided by Elizabeth M. Jaffee and Eric Lutz (Johns Hopkins University). NALM6 cells virally transduced with GFP and luciferase were provided by Marty Pomper (Johns Hopkins University). Isogenic cell line pairs were previously generated by CRISPR: p53 KO (KMS26, KLE, and TYK-nu) (5) or knockin (NALM6^R175H^) (37). Cells were cultured in complete media (10% FBS [Hyclone] and 1% penicillin-streptomycin [Gibco]). T2, RPMI-6666, T2A3, KMS26, SW620, and NALM6 cells were cultured in complete RPMI (cRPMI). RPMI-1640 (ATCC 30-2001) and KLE cells were cultured in DMEM F12 (ATCC 30-2006). TYK-nu and LS123 cells were cultured in EMEM (ATCC 30-2003). HCT116 cells were cultured in McCoy’s media (Gibco). HEK293FT cells were maintained in DMEM high glucose (4.5 g/L) (Gibco, 11965092) supplemented with 2 mM l-glutamine (Gibco), 0.1 mM nonessential amino acids (Gibco), and 1 mM sodium pyruvate (Gibco). All cells were cultured at 37°C with 5% CO_2_.

### Primary human T cells.

Primary human T cells were obtained from purchased leukopaks (StemCell Technologies). PBMCs were isolated by density gradient centrifugation using Ficoll-Paque Plus (Cytiva, 17144002) followed by ACK lysis (Quality Biological, 118-156-101).

For bispecific antibody experiments, T cells were expanded by OKT3 activation as previously described (4, 5, 34). Briefly, PBMCs were resuspended at 10^6^ cells/mL with 15 ng/mL anti-CD3 antibody clone OKT3 (BioLegend, 317325) in T cell media: cRPMI with 100 IU/mL recombinant human IL-2 (proleukin, Prometheus Laboratories) and 5 ng/mL recombinant human IL-7 (BioLegend, 581908). For bispecific antibody experiments, T cells were used 18 days after OKT3 activation.

For CAR T cells experiments, CD3^+^ T cells were isolated from PMBCs after Ficoll/ACK processing by negative selection (StemCell Technologies, 17951). Isolated CD3^+^ T cells were activated with Dynabeads Human T-Activator CD3/CD28 (Thermo Fisher Scientific, 11132D) at a 1:1 bead/cell ratio in T cell media for 48 hours.

### Peptides, monomers, and tetramers.

Peptides were purchased from Peptide 2.0 at >90% purity. Biotinylated monomers and tetramers were produced by the Fred Hutchinson Immune Monitoring Core (Fred Hutchinson Cancer Center). Monomer folding was confirmed by ELISA with antibody clone W6/32 as previously described (4, 5, 34).

### Enrichment from pooled phage with thiocyanate elution.

Ammonium thiocyanate elution was used to select strong binding H2 variants from the phage display library pool after 4 rounds of biopanning. EvenCoat streptavidin-coated microplates (R&D Systems) were coated with 0.1 μg/well biotinylated R175H/A2 monomer in BAE blocking buffer (PBS containing 0.5% BSA [Sigma-Aldrich], 2 mM EDTA [Thermo Fisher Scientific], and 0.1% sodium azide) and then washed with BAE. Precipitated phage was diluted 1:50 with BAE and applied to the plates in triplicate. After 1 hour, the plate was washed 6 times with TBST (J77500-K8, Thermo Fisher Scientific) followed by 4 times with HBS-PE (10 mM HEPES, 150 mM NaCl, 0.005% Tween 20, and 3 mM EDTA, pH 7–7.5) alone or HBS-PE containing 0.5 M NH_4_SCN, pH 7 (Sigma-Aldrich). The fourth wash was left on the plate overnight at 4°C on a plate shaker, and 2 final washes were completed the next day. Phage was eluted with 0.2 M glycine (pH 2.2), neutralized with Tris-HCl (pH 9), and then used to infect SS320-competent cells (in 2XYT media plus 1:1,000 helper phage, tetracycline 50 μg/mL, and 2% glucose) in 1 mL deep well plates (Nunc, Thermo Fisher Scientific). Bacteria were incubated for 1 hour at 37°C with shaking at 250 rpm (RCF = 0.67*g*), collected by centrifugation, and resuspended in 2XYT media containing 20 μM IPTG (Sigma-Aldrich), 100 μg/mL carbenicillin, and 50 μg/mL kanamycin. Plates were incubated overnight at 30°C and stored at 4°C for massively parallel sequencing as described in the Supplemental Methods.

Enriched variants were identified as variants with a frequency > fiftieth percentile and enrichment > 2 when comparing variant frequency in the thiocyanate elution condition to its frequency in the round 4 phage pool. Variants were ranked by frequency and enrichment to select candidates for functional testing. Y57I was included as a comparator from the previous monoclonal phage experiments.

### Bispecific antibody production.

Bispecific antibodies were produced in small batches for screening or purchased from GeneArt (Thermo Fisher Scientific) as previously described (4, 5). H2 variant plasmids were generated by site-directed mutagenesis (E0554S, New England Biolabs) in expression vector pcDNA3.4-TOPO (see Supplemental Table 3 for plasmid sequences). For double mutant scDbs, each light chain variant selected was paired with a heavy chain variant (e.g., V29E and Y57I). For small-batch scDb production, T75 flasks of HEK293FT cells were transfected with 20 μg plasmid DNA using the Lipofectamine 3000 kit (Thermo Fisher Scientific). After 4 days, scDbs were purified from the cell culture supernatant with HisPur Ni-NTA Resin (Thermo Fisher Scientific) with imidazole elution followed by buffer exchange into 20 mM Tris-HCl, pH 8.5–9, using 7K MWCO Zeba Spin desalting columns (Thermo Fisher Scientific). Large-scale production (2–5 liters) of scDbs were further purified by size-exclusion chromatography using GeneArt (Thermo Fisher Scientific). Bispecific antibody purity was confirmed by gel analysis. scDbs were quantified by gel (4%–20% Mini-Protean TGX Stain-Free Gels, Bio-Rad) or Pierce BCA Protein assay kit (Thermo Fisher Scientific).

### Bispecific antibody monomer ELISA.

scDbs were evaluated for binding to the p53 R175H/A2 monomer, p53 WT/A2 monomer (0.025 μg/well), or biotinylated CD3ε/CD3δ heterodimer (0.02 μg/well, Acro Biosystems) by ELISA as previously described (34); 5 ng/well scDb was added in triplicate. Plates were washed with TBST after 1 hour and incubated with 25 ng/well Pierce recombinant protein L (Thermo Fisher Scientific) for 1 hour. Plates were washed and incubated with 10 ng/well chicken anti–protein L HRP antibody (ab63506, Abcam) for 1 hour. Plates were washed, and bound antibody was detected with TMB substrate (BioLegend).

### Affinity determination by SPR.

scDb binding kinetics were determined by SPR using the Biacore T200 instrument (GE Healthcare, Georgetown University Biacore Molecular Interaction Shared Resource) as previously described (4, 5, 34). Binding responses were analyzed with Biacore Insight evaluation software using a 2-state binding model.

### Peptide-pulsing cocultures.

T2- or HLA-A3–expressing T2A3 cells at 10^6^ cells/mL were pulsed with varying concentrations of p53 WT or p53 R175H peptide in RPMI and washed prior to coculture. Pulsed target cells were incubated with primary T cells and 1 nM scDb or CAR T cells for 18–21 hours in 200 μL cRPMI containing 100 IU/mL IL-2. Cell numbers for individual experiments are listed in figure legends. For IFN-γ ELISA, 100 μL cell supernatant was frozen. Remaining cells were assayed for viability by CellTiter-Glo (Promega). Percent toxicity was calculated after subtracting the values from the T cell–only condition (no target cells) from each T cell condition. IFN-γ production was quantified with the Quantikine Human IFN-γ ELISA Kit (DIF50C, R&D Systems).

### Endogenous p53 mutant cell line cocultures.

scDb and CAR T cell activity was tested against luciferase-expressing cell lines containing an endogenous p53 R175H mutation or isogenic control (p53 KO or WT p53 [R175WT]) (5, 37). Individual experiment details are included in figure legends. All conditions used cRPMI containing 100 IU/mL IL-2. Target cell viability was assessed with the Steady-Glo Luciferase Assay System (Promega) or by flow cytometry.

### High content imaging cocultures.

TYK-nu target cells expressing Nuclight Green (Sartorius) were generated as described previously (5) for high-content imaging with the Incucyte SX5 instrument (Sartorius). Images were collected every 4 hours (4 images/well). Incucyte software was used to count Nuclight Green^+^ target cells or GFP^+^ NALM6 cells (identified by green fluorescence intensity) and to determine KLE cell confluency.

### Mouse experiments.

Female NSG mice were obtained from the Johns Hopkins Oncology Research Animal Resources program and maintained in compliance with the IACUC-approved protocol (MO21M43). For the KMS26 delayed treatment model, NSG mice age 7–9 weeks were inoculated with 3.5 × 10^5^ luciferase-expressing KMS26 cells by tail vein injection 7 days prior to treatment day. On treatment day –1, tumor burden was measured by bioluminescent imaging using the IVIS Spectrum In Vivo Imaging system (PerkinElmer), RediJect D-Luciferin Ultra Bioluminescent Substrate (PerkinElmer, 770505) and quantification with Living Image software (PerkinElmer). Mice were randomized into 3 treatment arms: H2^29.5^, H2^6.8^, or isotype control (scDb to an unrelated pHLA) (*N* = 5 per group). On treatment day 0, mice received 10^7^ primary human T cells i.v. scDb treatment was delivered by continuous elution for 14 days with surgically implanted intraperitoneal micro-osmotic pumps (ALZET, 1002). scDb was dosed at 0.075 mg/kg/d (1.5 μg/d).

For the early treatment model with NALM6^R175H^, NSG mice age 8–10 weeks were inoculated with 5 × 10^5^ luciferase-expressing NALM6^R175H^ cells and 10^7^ primary human T cells by tail vein injection. Two days later, mice were treated with scDb and imaged as described above.

### CAR HDRT design and generation.

Plasmid sequences for the AV6/BV11 TCR, CD3γ TRuC CAR, STAR, and second-generation CD28-CD3ζ (H2-CD28z) CARs were previously described (37) (see Supplemental Table 3). The CD28z CAR employs a CD8α hinge, CD28 transmembrane and intracellular domain, and CD3ζ intracellular domain (37, 43). TRuC consists of the H2 scFv appended to the CD3γ subunit of the TCR complex. Both H2-CD28z CAR and H2-CD3γ TRuC were designed for CRISPR knockin at the *CD3G* locus. The patient-derived TCR AV6/BV11 and H2 STAR were designed for CRISPR knockin at the *TRAC* locus with simultaneous *TRBC* KO and used modified murine TCRα and TCRβ constant domains to discourage mispairing with endogenous human TCR (46). Plasmid sequences are listed in Supplemental Table 3.

Double-stranded DNA homology-directed repair templates (HDRTs) were generated by PCR amplification using the Q5 Hot Start High-Fidelity 2X Master Mix (New England Biolabs) and the following primers: M13_tCTS_F, TGGCGGGACTAGTGGCAGAGCTACCTTTGATTGACTGGGCAGTCACGACGTTGTAAAACG; M13_tCTS_R, CACCACTTCCAGCACCAGAGCTACCTTTGATTGACTGGGAGCGGATAACAATTTCACACAGG. HDRTs were purified with 1× AMPure XP reagent (Beckman Coulter Life Sciences, A63880) and eluted in sterile water.

### CRISPR editing of primary human T cells.

CRISPR editing was performed as previously described (37). Briefly, T cells were separated from CD3/CD28 beads after 48–56 hours. For 10^6^ cells, ribonucleoproteins (RNPs) were prepared by combining 100 pmol Alt-R A.s. Cas12a crRNA (IDT), 50 pmol Alt-R A.s. Cas12a (Cpf1) Ultra (IDT), and 75 pmol of Alt-R Cpf1 electroporation enhancer (IDT) in a total volume of 2.54 μL in Nuclease Free Duplex Buffer (IDT). Each 1× electroporation reaction consisted of 2.54 μL of RNPs and 0.5 μg HDRT in 2.4 μL OptiMEM (Gibco). The 10^6^ T cells in 20 μL P3 buffer (Lonza, V4XP-3032) were added to the 5 μL RNP-HDRT mixture. Cells were nucleofected in 16 well cuvettes (Lonza, V4XP-3032) with a 4D Nucleofector X-Unit (Lonza, AAF-1003X) using pulse code EH115. Eighty microliters of prewarmed cytokine-free cRPMI was then added to each well. After 20 minutes of rest at 37°C, cells were transferred to 24-well plates. T cell media was changed on days 3, 6, and 9. On days 10–12, cells were purified by positive selection for NGFR-expressing cells (StemCell Technologies, 100-0047) and used for functional experiments on day 11 or after. T cell editing was determined by flow cytometry after purification. For all functional experiments, T cell conditions were normalized to the lowest percent edited cells using excess TRAC/TRBC-KO cells (minimum across experiments was >75% edited T cells). All gRNA sequences used are listed in Supplemental Table 4.

### Flow cytometry.

Flow cytometry was performed with the IntelliCyt iQue Screener PLUS (Sartorius), Attune NXT (Thermo Fisher Scientific), or CYTEK Aurora (Cytek Biosciences). Cells were stained with LIVE/DEAD Fixable Dead Cell Stain (Invitrogen). For characterization of CAR T cell editing, cells were stained with combinations of CD3 (SK7), CD4 (RPA-T4), CD8 (RPA-T8), murine TRBC1 (H57-597), R175H/A2 tetramer (Fred Hutchinson), and NGFR (ME20.4).

For flow analysis of cocultures, 10^4^ Precision Plus Count Beads (BioLegend) were added to each well prior to staining with LIVE/DEAD Fixable Dead Cell Stain (Invitrogen) and combinations of HLA-DR (LN3), NGFR (ME20.4), mTRBC1 (H57-597), CD25 (BC96), CD69 (FN50), and CD3 (SK7).

For intracellular cytokine staining, cocultures were conducted in the presence of 1× brefeldin A (BioLegend) and 100 IU/mL IL-2 for 17 hours. Counting beads were added, and cells were stained with LIVE/DEAD followed by antibodies for CD4 (RPA-T4) and CD8 (RPA-T8). Cells were permeabilized with Cyto-Fast Fix/Perm buffer set (BioLegend) and stained with CD137 (4B4-1), CD69 (FN50), and IFN-γ (4S.B3).

All antibodies were obtained from BioLegend. Flow data were analyzed using IntelliCyt iQue software (Sartorius) or FlowJo version 10.

### MSA.

T cells were stained with CellTrace Violet (Invitrogen) for proliferation tracking. On day 0 of the MSA, 4 × 10^3^ edited T cells were cocultured with 1.6 × 10^4^ GFP^+^ target cells. On days 2, 4, and 6, replicate plates were either rechallenged with 3.2 × 10^4^ target cells or assayed by flow cytometry (after removing 100 μL supernatant for IFN-γ ELISA). On days 0, 2, 4, and 6, 100 IU/mL IL-2 was added. Proliferation analysis was performed using FlowJo version 10.

### Live-cell imaging.

KLE target cells were plated approximately 18 hours prior to imaging: 2.5 × 10^4^ cells/well in a tissue culture–treated #1.5 glass-like polymer-based 24 well plate (CellVis, P24-1.5P). Edited T cells were brought to 2.93 × 10^5^ cells/mL in cRPMI containing 100 IU/mL IL-2. For a 6× reaction, collagen matrix components were gently mixed on ice in order: 120 μL 10× MEM (Sigma-Aldrich, M0275), 60 μL 7.5% sodium bicarbonate (Gibco), and 888 μL 3 mg/mL PureCol-S bovine collagen (Sigma-Aldrich, CC300) (61). After 5 minutes on ice, 50 μL was removed for pH measurement, and 552 μL T cells was added to the collagen matrix immediately prior to addition to the 24-well plate. Culture media was aspirated from the KLE-plated wells, and 350 μL collagen-cell mixture was added dropwise to each well (3.5 × 10^4^ T cells). Plates were incubated for 30 minutes at 37°C, 5% CO_2_ for polymerization, and 650 μL prewarmed cRPMI containing 100 IU/mL IL-2 was added to each well. Plates were transferred to the microscope incubation chamber (Tokai, humidified 5% CO_2_, 37°C). Imaging began 1 hour after the start of polymerization.

T cell killing of KLE target cells was observed using a 3i Marianis/Yokogawa Spinning Disk confocal microscope with a Zeiss AxioObserver.Z1 inverted microscope, Hamamatsu Orca Flash 4.0LT high-speed sCMOS camera, and 3i SlideBook software. Bright-field images were captured every 30 seconds at 20× magnification with 1 × 1 binning at 12–15 nonoverlapping positions across a single well. Each T cell condition was evaluated in 3 independent 18–22 hour experiments. Deidentified images were analyzed in Fiji (ImageJ) using the Trackmate manual cell tracking plug-in. T cells were selected for manual tracking if a single T cell bound to a KLE target cell followed by target cell blebbing (only 1 T cell interaction per target cell killing event). Time to blebbing was calculated from time of stable T cell interaction with a target cell (T cell rounding, decreased motion) (62). For T cells adhered to a KLE target cell at the start of imaging, 1 hour was added to the time for blebbing to account for the 1 hour interval to start of imaging. Time to T cell detachment was calculated from time of blebbing.

### Statistics.

All data are presented as mean ± SD unless otherwise specified. Statistical tests used are indicated in figure legends. A *P* value less than 0.05 was considered significant. Prism 10 (GraphPad) was used for data analysis, graphing, and statistical analysis.

### Study approval.

Animal studies were reviewed and approved by the Johns Hopkins Medical Institutions Oncology Research Animal Resources program and IACUC under protocol MO21M43.

### Data availability.

All data associated with this study are present in the paper or the Supporting Data Values file. Sequencing data files are available on Dryad at https://doi.org/10.5061/dryad.vdncjsz83 Plasmids and cell lines are available by request under a material transfer agreement with Johns Hopkins University by contacting SZ.

## Author contributions

SRD, KWK, BV, and SZ conceived the study. SRD, BJM, EHCH, JD, MSH, JDC, MP, KWK, and BV developed the methods. SRD performed the in vitro experiments. JDC, MP, and KWK assisted with next-generation sequencing design and analysis. KMW assisted with affinity measurements. SRD, EW, QL, MDM, and KLG performed the in vivo studies. SRD, KMW, BJM, AHP, TDN, NM, SP, MFK, NW, PAA, SG, JG, TOA, JL, KLG, SBG, DMP, CB, NP, KWK, SZ, and BV assisted with analysis and interpretation of data. SRD, BV, and SZ wrote the original draft. SRD, BJM, SP, TDN, NM, JD, JG, NW, MDM, KLG, SZ, and BV reviewed and edited the final manuscript. SZ, KWK, and BV supervised the study.

## Funding support

This work is the result of NIH funding, in whole or in part, and is subject to the NIH Public Access Policy. Through acceptance of this federal funding, the NIH has been given a right to make the work publicly available in PubMed Central.

- The Virginia and D.K. Ludwig Fund for Cancer Research (to BV, NP, KWK, and SZ).
- The Lustgarten Foundation for Pancreatic Cancer Research (to BV, NP, KWK, and SZ).
- The Commonwealth Fund (to BV, NP, KWK, SZ, and CB).
- Bloomberg Philanthropies and the Bloomberg~Kimmel Institute for Cancer Immunotherapy (to DMP, BV, KWK, and SZ).
- NIH Cancer Center Support grant P30 CA006973 (to KWK and NP).
- NIH grant T32 GM136577 (to BJM, SRD, JD, AHP, TOA, and JDC).
- NIH grants T32 AR048522 and 1R21 AI176764 (to MFK).
- NIH grant K08CA270403 (to SP).
- NIH grant R21 HL156224 (to KLG).
- NIH National Institute of General Medical Sciences grant T32GM148383 (to NM).
- NIH grant P30CA51008 to the Biacore Molecular Interaction Shared Resource (BMISR).
- National Cancer Institute (NCI) grant T32 CA153952 (to TDN).
- NCI grant T32 CA126607 (to MDM).
- NCI grant R37 CA230400 (to CB).
- NCI grant K08CA270403 (to SP).
- Awards from the Leukemia Lymphoma Society Translation Research Program (to SP).
- American Society of Hematology Scholar (to SP).
- Swim Across America Translational Cancer Research (to SP).
- The Jerome Greene Foundation (to MFK).
- The Cupid Foundation (to MFK).
- The Stephen and Renee Bisciotti Foundation (to MFK).
- The Harrington Scholar-Innovator Grant (to MFK).
- The Rheumatology Research Foundation (to MFK).

## Supplementary Material

Supplemental data

Supplemental table 3

Supporting data values

## Figures and Tables

**Table 1:**
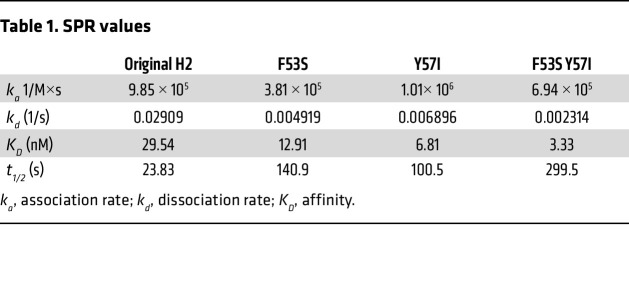
SPR values

**Table 2:**
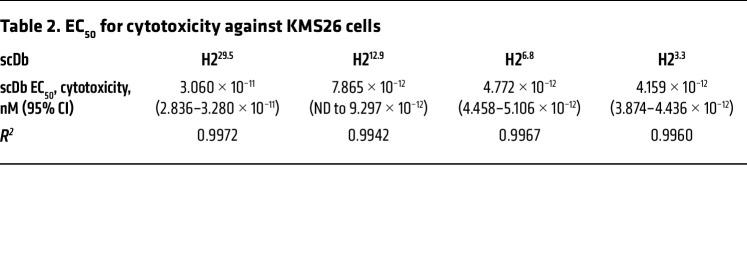
EC_50_ for cytotoxicity against KMS26 cells

**Table 3:**
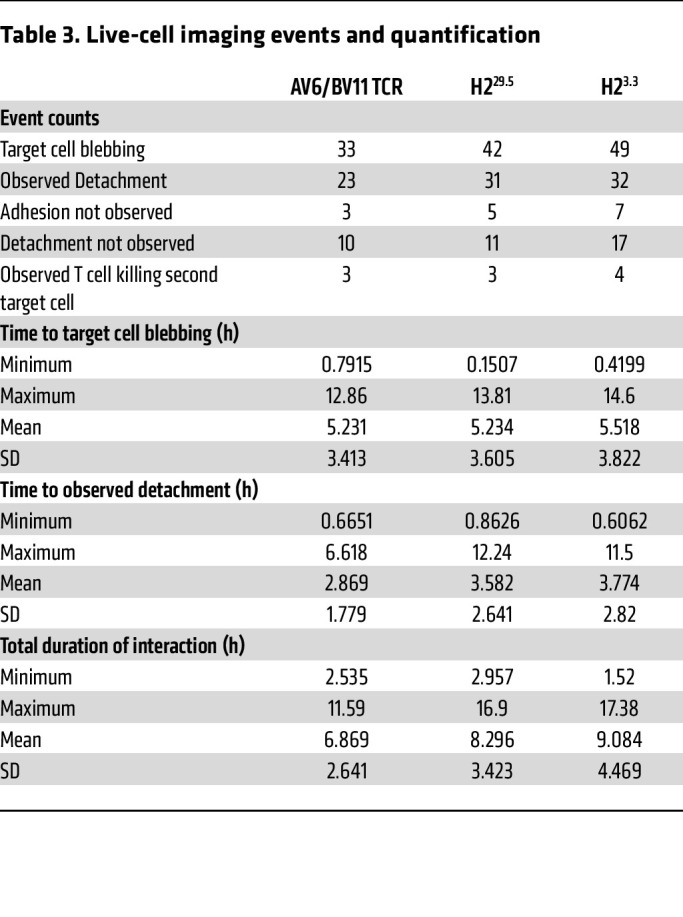
Live-cell imaging events and quantification

## Version 1 01/16/2026

Electronic publication
