# Supplementary material for: Bispecific antibodies and CAR T cells targeting a TP53 mutation–associated neoantigen show discordant affinity requirements
Source: J Clin Invest. 2026 Jan 16;136(2):e192885. doi: 10.1172/JCI192885 (PMC12807468; doi:10.1172/JCI192885)
Supplement: Supplemental data [file jci-136-192885-s207.pdf]

Supplemental Materials

Contents

Supplemental Methods

Supplemental Figures:

S1. H2 monoclonal phage ELISA

S2. H2 scFv phage display library screening and sequencing

S3. Bispecific antibody characterization

S4. Interferon gamma response to endogenous antigen levels.

S5. Higher affinity H26.8 has improved tumor control in vivo.

S6. CAR T cell constructs at the CD3G locus

S7. Higher affinity 2nd generation CARs and CD3γ-TRuC T cells are less sensitive

S8. STAR binding to R175H/A2 tetramer

S9. Multiple stimulation assay

S10. STAR T cell killing of KLE target cells

S11. CD25 and CD69 upregulation in TCR CAR and scDb co-cultures

S12. Evaluating STAR exhaustion after multiple stimulation assay

Supplemental Tables:

S1. H2 scFv library variant frequencies determined by Twist Bioscience.

S2. R175H peptide EC50s for H2 variant bispecific antibodies

S3. Plasmid sequences (see separate data file)

S4. CRISPR Guide RNAs

Supplemental Methods

Affinity maturation phage display library design

A library of H2 variants was designed as previously described(1). Briefly, H2 scFv sequence was annotated using the AbYsis tool(2) to identify complementarity determining regions (CDRs). Variant sites were selected to include CDRs and neighboring residues with unusual amino acids (low frequency in *Homo sapiens* sequences at that site per AbYsis) and exclude sites with low diversity ($\leq$3 total amino acids representing >90% of *H. sapiens* sequences in the AbYsis database). 61 sites were selected for modification to the other 19 amino acids for a total of 1159 H2 variants. The variant library was synthesized and cloned into the pADL-10b phagemid vector (Antibody Design Labs, San Diego, CA) by Twist Biosciences (San Francisco, CA). Sequence diversity was confirmed by massively parallel sequencing by Twist Bioscience (see Supplemental Table 1).

Phage display library biopanning

The H2 variant phage display library was screened by biopanning as previously described(1, 3–5). Briefly, a reference library was produced by electroporating SS320 competent cells with the H2 library plasmid DNA and stored in glycerol at -80˚C. Electroporated bacteria were infected with M13K07 helper phage (Antibody Design Labs, San Diego, CA) to produce a starting phage library (titer 1.25×10^{11} cfu/ml). For negative selection in Round 1 of panning, the phage library was incubated with uncoated streptavidin M-280 Dynabeads (ThermoFisher Scientific, Carlsbad, CA) and streptavidin (Invitrogen, Camarillo, CA) overnight at 4˚C. Supernatant was applied to M280 beads coated with 1 µg biotinylated R175H/A2 monomer for positive selection and incubated for 1 h at room temperature. Beads were washed 10 times with TBST. Phage was eluted with 0.2 M glycine pH 2.2, neutralized with Tris HCl pH 9 and used to

infect 10 ml SS320 competent cells along with M13K07 helper phage (MOI 4). After 1 h incubation at 37˚C, infected SS320 cells were pelleted and resuspended in 2XYT media containing 20 µM IPTG (Sigma-Aldrich, St. Louis, MO) and incubated overnight at 30°C for phage production. Phage was precipitated using a 4:1 ratio of phage to PEG/NaCl, isolated by centrifugation and resuspended in TBS/EDTA/0.1% sodium azide. For subsequent rounds of negative and positive selection, phage was incubated with HLA-A2 positive, p53 R175H-negative cell lines (SW620, RPMI-6666, HCT116, T2A3, HEK293FT, LS123, KMS26 p53 knockout clone) and then M280 beads coated with HLA-A2 monomers presenting p53 WT peptide (HMTEVVRRC) or unrelated peptides for negative selection followed by positive selection with p53 R175H/A2 monomer-coated beads. After each round of positive selection, eluted phage was used to infect SS320 cells to produce high titer phage for subsequent rounds of panning.

Monoclonal Phage selection

Monoclonal phage clones were selected from the panning round 4 and round 5 pools as previously described(1, 3, 4). The H2 Fab-R175H/A2 crystal structure (Protein Data Bank ID: 6W51) and PyMOL Version 2.4 (Schrödinger, LLC) were used to to identify 19 variants with potential changes in hydrogen bonding, hydrophobic interactions or proximity to the R175H side chain. For example, A31S^{CDRH1} was predicted to allow for formation of a new hydrogen bond while Y57I^{CDRH2} could enhance a hydrophobic "cage" around the R175H histidine side chain

Monoclonal Phage ELISA

To test phage binding to mutant vs. wild-type monomers, streptavidin-coated plates (R&D Systems, Minneapolis, MN) were coated with 50 µl of 0.5 µg/ml biotinylated R175H/A2 monomer or R175WT/A2 in BAE buffer (PBS containing 0.5% bovine serum albumin (Sigma),

2 mM EDTA (Thermo) and 0.1% sodium azide) for 1 h at room temperature. Plates were washed 6 times with 1X TBST (J77500-K8, Thermo Fisher Scientific) using a 405 TS microplate washer (BioTek, Winooski, VT). Phage-laden supernatant from the single colony cultures was diluted 8-fold with 1X TBST and added to the monomer-coated plates for a 2 h incubation at room temperature. After 2 h, the plates were washed 6 times with 1X TBST. Plates were then incubated for 1 h at room temperature with a polyclonal rabbit anti-fd/M13 bacteriophage antibody (NB100-1633, Novus, Centennial, CO) diluted 1:5000 in 1X TBST. Plates were washed 6 times with 1X TBST. Plates were then incubated with goat anti-rabbit IgG (H + L) Secondary (HRP) antibody (NB7160, Novus, Centennial, CO) diluted 1:10,000 in 1X TBST for 1 h at room temperature. Plates were washed 6 times with 1X TBST. Bound phage was detected by adding 3,3′,5,5′-tetramethylbenzidine (TMB) substrate (BioLegend) and quenching the color formation reaction with 1 N sulfuric acid (Fisher Scientific). Absorbance was measured at 450 and 540 nm using a Synergy H1 Multi-Mode Reader (BioTek).

Massively parallel sequencing

We initially screened monoclonal phage colonies identified by Sanger sequencing(3, 4). To better assess library diversity and enrichment, we adapted an Illumina MiSeq system to allow for multi-read sequencing of the ~700 bp scFv by massively parallel sequencing. scFv sequences were amplified from eluted phage or phage-laden media using the following primers (5’-3’):

CDRL1-Amp-F (including N14 UID):

CGACGTAAAACGACGGCCAGTNNNNNNNNNNNNNNGCAAGCGTTGGTGATCGTGTT ACCATTACC, Amp-Rev:

CACACAGGAAACAGCTATGACCATGCTGCTAACGGTAACCAGGGTGC. PCR amplification was performed in 25 μl reactions using NEBNext Ultra II Q5 master mix (New

England Biolabs, Ipswich, MA) with 5 μM primers and 2.5 μl template with the following cycles: 98°C 2 min, then 20 cycles: 65°C 30 s, 72°C 20 s, then 72°C 2 min, hold 4°C. Library preparation was performed as previously described with 5-10 cycles of amplification(6). Samples were evaluated with 8 sequencing reads [Primer name, sequence, # of read cycles]: SafeSeqS_fwd, CGACGTAAAACGACGGCCAGT, 86; CDRL3_Fwd, GCAGCCGGAAGATTTGCAACCTATTATTGC, 32; CDRH3_Fwd, GCGTGCAGAGGATACAGCAGTGTATTATTG, 40; CDRH1_Fwd, GGTGGTAGCCTGCGTCTGAGC, 44; CDRH2_Fwd, GGCACCTGGTAAAGGTCTGGAATGG, 40; CDRL2_Fwd, CAGCAGAAACCGGGTAAAGCACCG 37; Index, ACTGGCCGTCGTTTTACGTCG, 8. Sequencing results were identified by index and UID as previously described(6, 7). Trimmed DNA sequences were translated into amino acids using Python (version 3.7.17) in JupyterLab notebook (version 1.2.6) via Anaconda3 Navigator (version 1.9.12). Only sequences with zero or 1 amino acid changes were included for variant frequency and enrichment analysis.

scDb differential scanning fluorimetry

scDb stability was assessed by differential scanning fluorimetry (DSF) as previously described (3). 20 μl solutions containing 2 μg scDb and 5X SYPRO Orange dye (Thermo Fisher Scientific) in PBS were aliquoted into a skirted clear 96-well PCR plate. Melt curve testing was performed using a QuantStudio 3 Real-Time PCR system (Applied Biosystems) from 25-100°C with 1°C/min steps. The negative first derivative of fluorescence vs. temperature (-d (fluorescence)/dT) was determined using the QuantStudio software (Applied Biosystems, v1.5.1). ScDb melting temperatures (T_m) were identified as the temperature at which the average negative first derivative was at its minimum for each scDb (n=3).

scDb Solubility testing

scDbs were freshly thawed and quantified by Pierce BCA protein assay kit (Thermo Fisher Scientific) then brought to 500 ng/ml (10 nM) in 20 mM Tris-HCl 150 mM NaCl pH 9. Samples were incubated overnight at 4°C. 10 µl aliquots were reserved for future quantification. Remaining sample was concentrated using 10 kDa Amicon Ultra-4 centrifugal filter units (Millipore Sigma) to 10X lower volume. Remaining sample was spun at 18,000 rcf at 4°C for 20 minutes to assess for precipitates. No precipitates were observed. A 2 µl sample was diluted with 40ul BCA assay reagent for quantification. BCA assay performed with 3 technical replicates per condition.

References

1. Wright KM, et al. Hydrophobic interactions dominate the recognition of a KRAS G12V neoantigen. *Nat Commun*. 2023;14(1):5063.

2. Swindells MB, et al. abYsis: Integrated Antibody Sequence and Structure—Management, Analysis, and Prediction. *J Mol Biol*. 2017;429(3):356–364.

3. Douglass J, et al. Bispecific antibodies targeting mutant RAS neoantigens. *Sci Immunol*. 2021;6(57):eabd5515.

4. Hsiue EH-C, et al. Targeting a neoantigen derived from a common TP53 mutation. *Science*. 2021;eabc8697.

5. Skora AD, et al. Generation of MANAbodies specific to HLA-restricted epitopes encoded by somatically mutated genes. *Proc National Acad Sci*. 2015;112(32):9967–9972.

6. Kinde I, et al. Detection and quantification of rare mutations with massively parallel sequencing. *Proc Natl Acad Sci*. 2011;108(23):9530–9535.

7. Lu S, et al. The rapid and highly parallel identification of antibodies with defined biological activities by SLISY. *Nat Commun*. 2023;14(1):17.

Supplemental Figure 1.

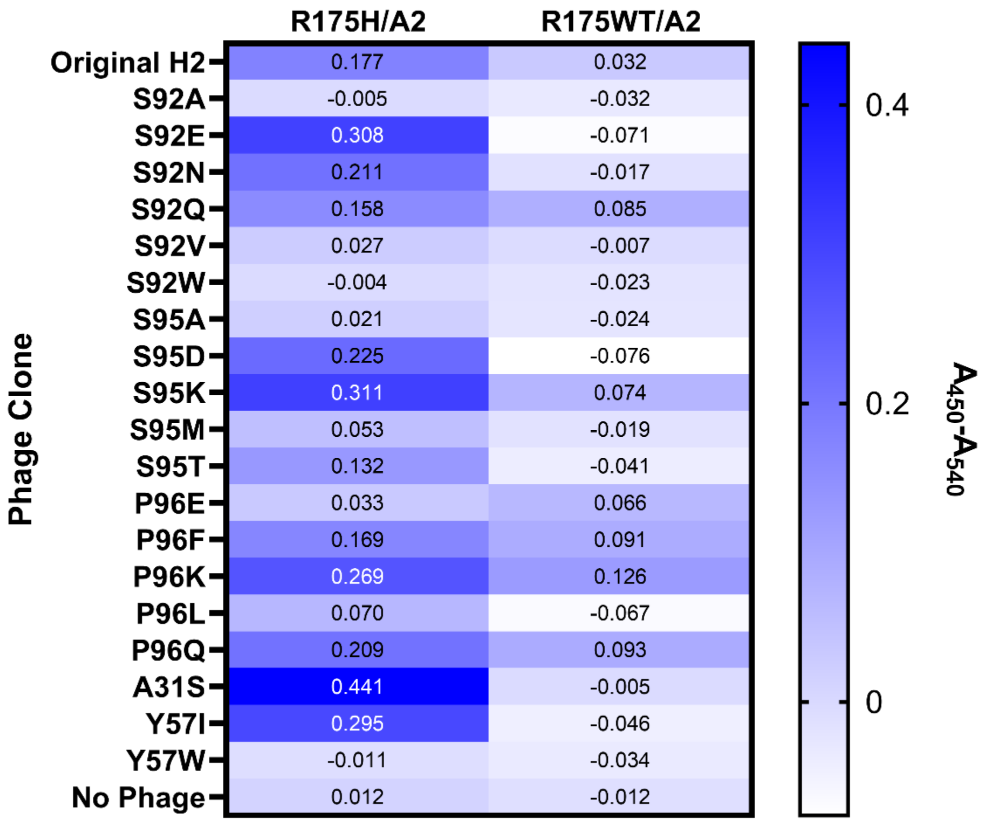

Supplemental Figure 1. H2 monoclonal phage ELISA. 19 H2 variant phage clones were selected for proximity to the R175H residue or role in hydrogen bonding in the crystal structure of H2 Fab bound to R175H/HLA-A2. Phage were tested for specific binding to the p53 R175H/A2 monomer compared to the R175WT/A2 monomer by ELISA. Absorbance at 450 nm was corrected by subtraction of absorbance at 540 nm. Data shown are single replicates from a single experiment.

Supplemental Figure 2.

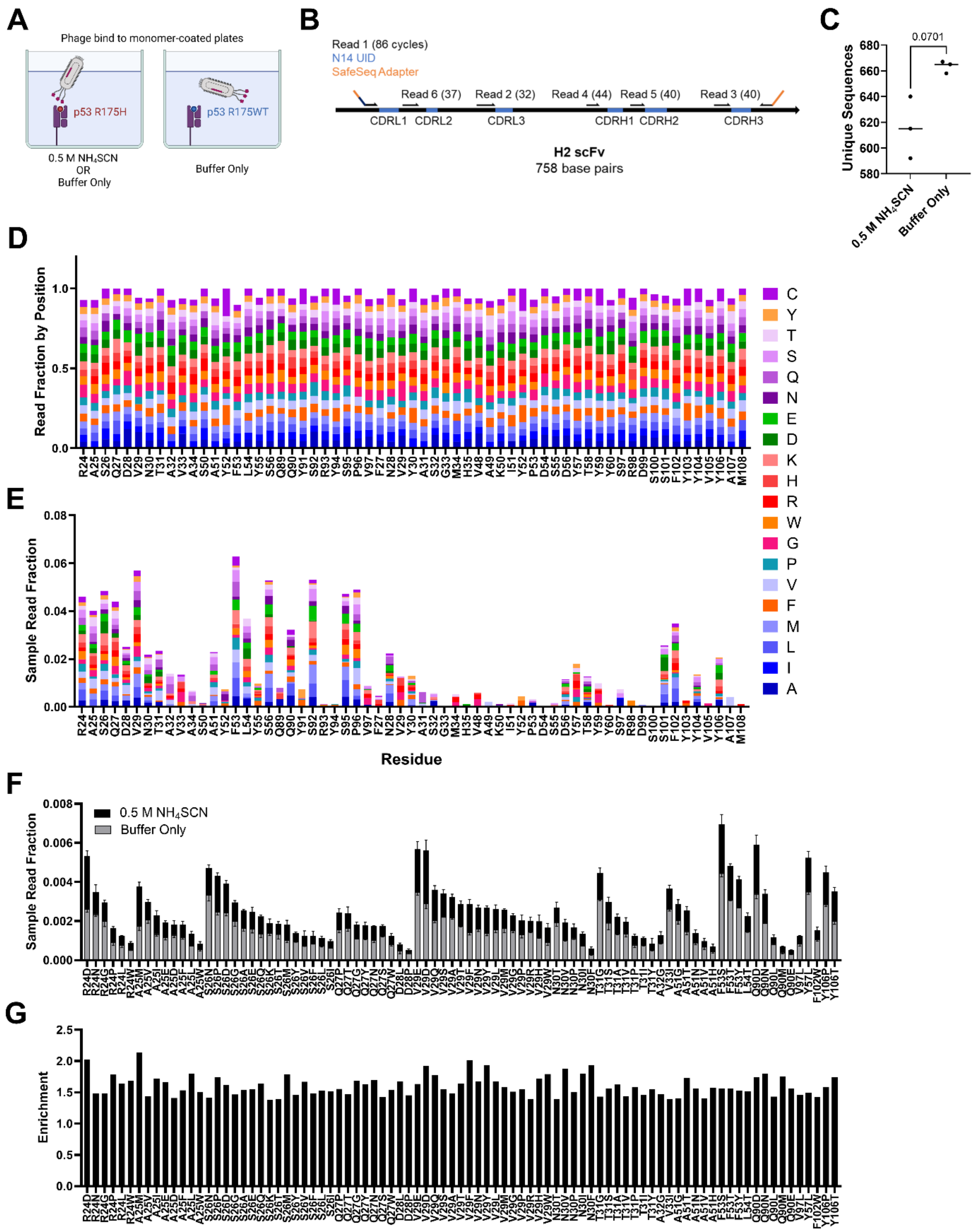

Supplemental Figure 2. H2 scFv phage display library screening and sequencing. (**A**) Pooled phage from the end of 4 rounds of panning was applied to microtiter plates coated with p53 R175H/A2 or p53 R175WT/A2 monomer and then washed overnight with either buffer alone or 0.5 M NH_4SCN in buffer (R175H condition only). (**B**) scFv sequences were PCR amplified from eluted phage and sequenced with 6 sequencing primers and 2 index reads. PCR-amplified products contain a 14-base pair unique identifier (N14 UID) and M13 adaptor sequences compatible with SafeSeqS library preparation. (**C**) After thiocyanate treatment, 615.7 +/- 24.0 unique variants remained compared to 663.3 +/- 4.7 variants in the buffer only condition. Means were compared by Welch's t test (n= 3 technical replicates, single experiment). (**D**) Variant read fraction by position in the starting H2 variant library determined by next generation sequencing at Twist Biosciences. (**E**) Variant read fraction after 4 rounds of negative and positive selection. Variant amino acids are colored by group: aliphatic hydrophobic (blue), aromatic hydrophobic (orange), proline (teal), glycine (pink), basic charged (red), acidic charged (green), polar neutral (purple). (**F**) Average read fractions of top variants after thiocyanate enrichment (black bars) or buffer-only treatment (gray bar) (n=3 technical replicates per condition). Variants with a read fraction >50th percentile in the thiocyanate condition and above average enrichment over the buffer-only condition are displayed. (**G**) Enrichment values for the variants in (**F**) were calculated by dividing the average read fraction in the thiocyanate condition by the average read fraction in the buffer-only condition.

Supplemental Figure 3.

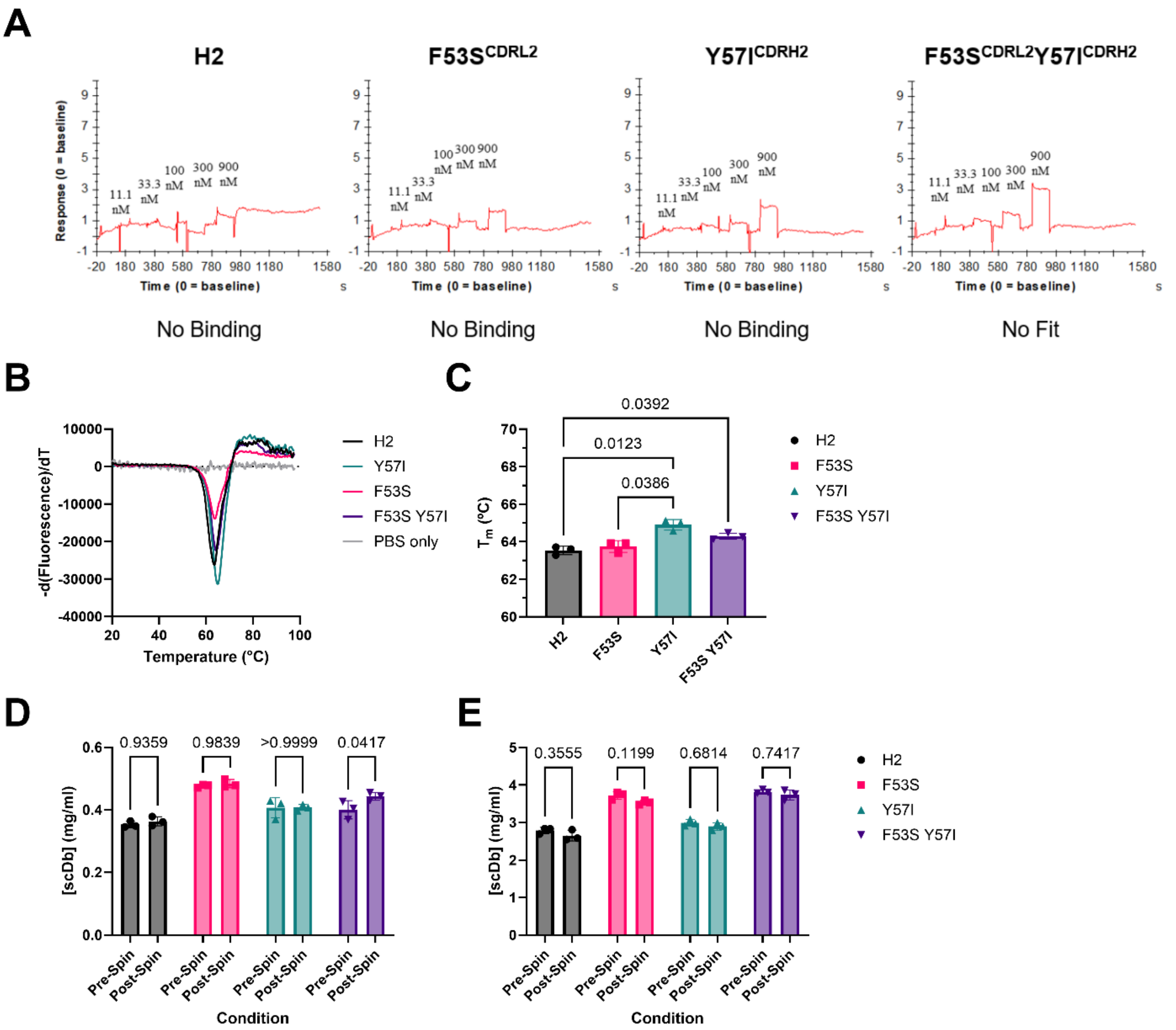

Supplemental Figure 3. Bispecific antibody characterization. (**A**) Single-cycle binding kinetics for each scDb binding to the p53 R175WT/HLA-A*02:01 monomer measured by SPR. Flow cell ratio 2:1. No fit = KD value could not be determined via curve fitting due to data quality. (**B-C**) Bispecific antibody melt temperature (T_m) was measured by differential scanning fluorimetry with SYPRO Orange. (**B**) The first derivative of fluorescence vs. temperature is plotted (dF/dT), n = 3 per scDb. (**C**) Mean ± SD melt temperatures for each bispecific antibody are plotted: 63.54, 63.75, 64.91, 64.29. T_ms for each bispecific were compared by one-way ANOVA with Brown-Forsythe and Welch's correction followed by Dunnett's T3 multiple comparisons test, n = 3 per scDb. Bispecific antibody concentration in the soluble fraction (supernatant) was measured by bicinchoninic acid (BCA) assay at low concentration (**D**) and after 10-fold concentration (**E**). Concentrations were compared by two-way ANOVA with Šídák's multiple comparisons test (n = 3 technical replicates per condition, single experiment).

Supplemental Figure 4.

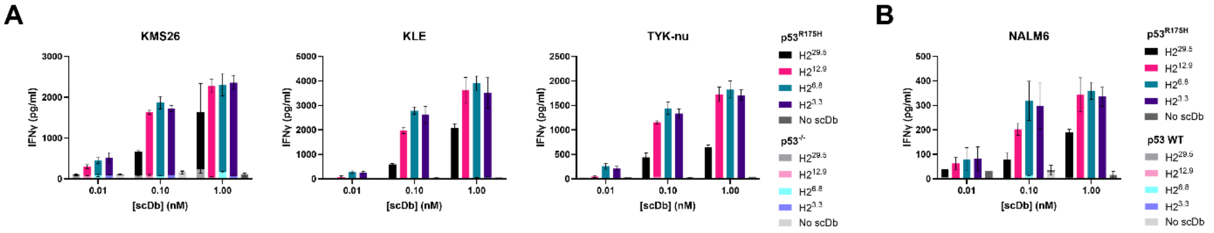

Supplemental Figure 4. Interferon gamma response to endogenous antigen levels. 5 x 10^4 human T cells were co-cultured with 2.5 x 10^4 luciferase-positive target cells in the presence of 0, 0.01, 0.1 or 1 nM scDb for 20 h. IFN-γ production was measured by ELISA for co-cultures with isogenic *TP53*^{R175H} and p53$^{-/-}$ cell lines (**A**) or p53^{R175WT} cell lines (**B**). Mean ± SD, n = 3, single experiment**.**

Supplemental Figure 5.

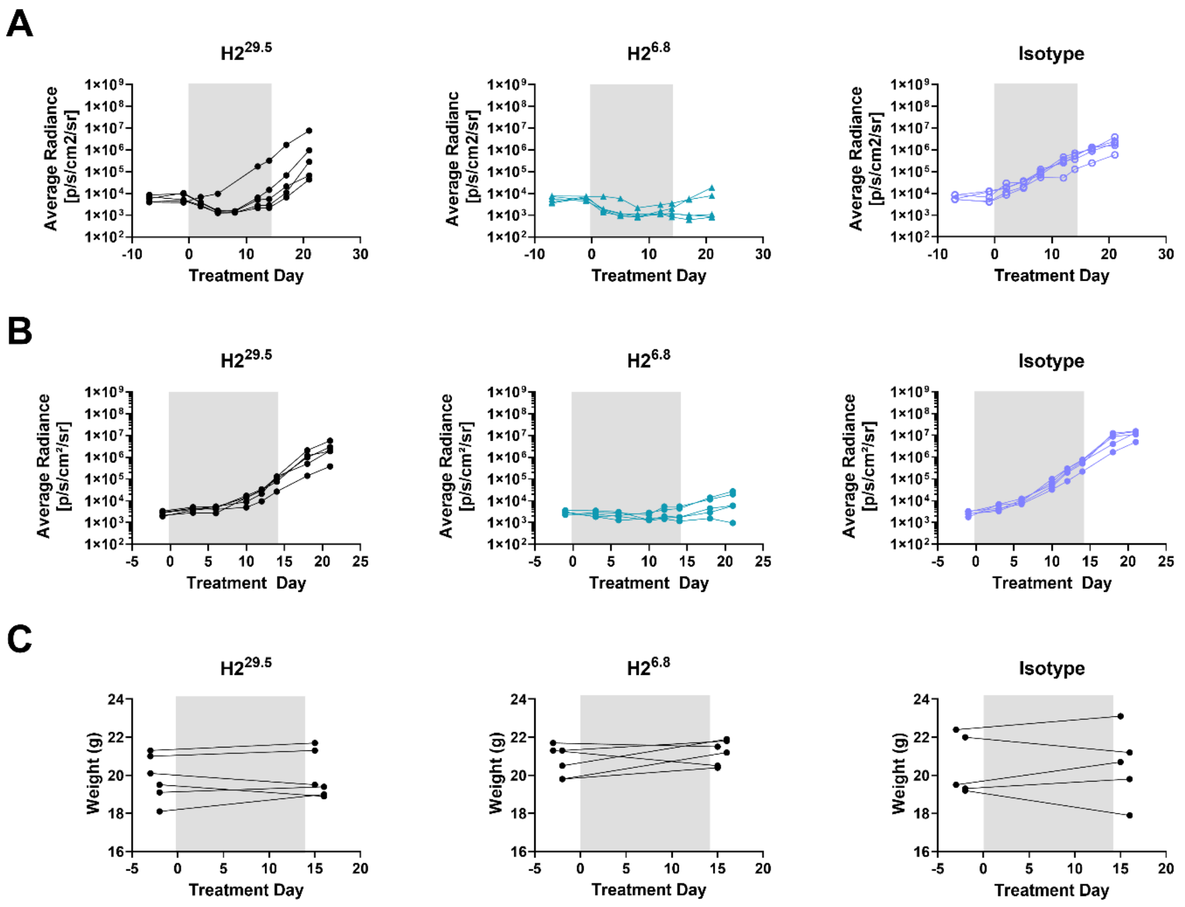

Supplemental Figure 5. Higher affinity $H2^{6.8}$ has improved tumor control in vivo. (**A**) Individual mouse tumor burden measurements in the delayed treatment model (KMS26 cell line). Treatment window is indicated by gray box. n = 5 per group. (**B**) Individual mouse tumor burden measurements in the early treatment model (NALM6 cell line). Treatment window is indicated by gray box. n = 5 per group. (**C**) Body weights for NSG mice inoculated with 3.5 x 10^5 KMS26 cells i.v. 7 days prior to treatment start. On treatment day 0, mice received 10^7 human T cells i.v. and surgically-implanted continuous release pumps i.p.. scDb was dosed at 0.075 mg/kg/d for 14 days. n = 5 per group.

Supplemental Figure 6.

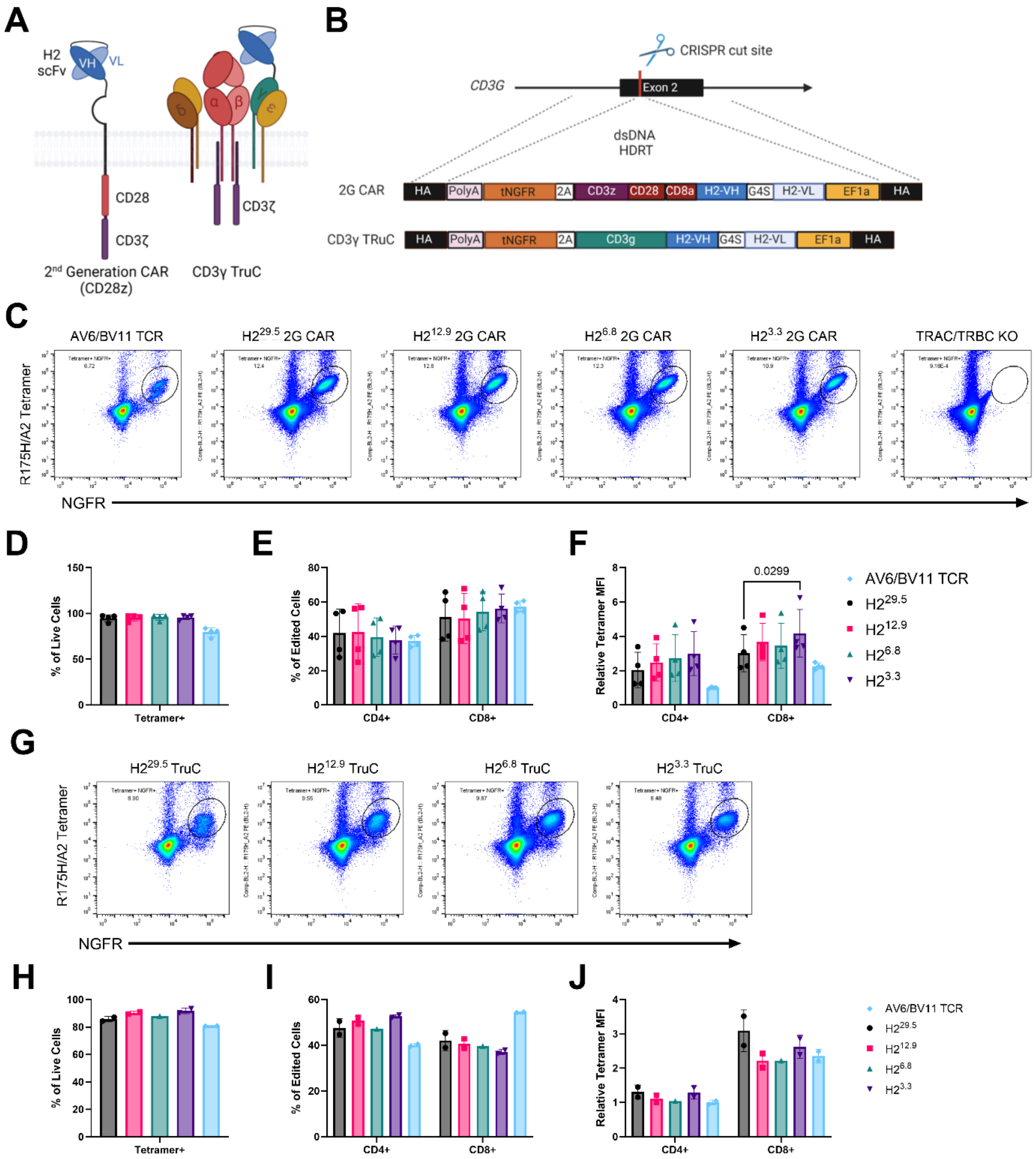

Supplemental Figure 6. CAR T cell constructs at the *CD3G* locus. (**A**) CAR and CD3γ-TRuC designs. 2nd generation CAR with the H2 scFv appended to a CD8α hinge, CD28 transmembrane and intracellular domain, and CD3ζ intracellular domain. (**B**) CAR T cells were generated by CRISPR knock-in at the *CD3G* locus. dsDNA homology directed repair templates (HDRTs) were generated by PCR for the 2nd generation CAR constructs (2G CAR) or CD3γ TRuC constructs. HA – homology arm; tNGFR – truncated nerve growth factor receptor; H2-VH – H2 variable heavy chain; H2-VL – H2 variable light chain. (**C**) Representative flow staining for 2G CARs and AV6/BV11 TCR. Human T cells were stained with LiveDead Near-IR (NIR), R175H-

HLA-A2 tetramer APC and anti-NGFR PE to determine CRISPR editing efficiency. Live singlets were gated for tetramer and NGFR expression. CRISPR edited CAR T cells are NGFR+ Tetramer+. (**D**) Editing efficiency was assessed by flow cytometry on days 11-13 with R175H/A2 tetramer staining. (**E**) CD4 and CD8 T cell populations were quantified by flow cytometry. (**F**) Tetramer staining on CD4 and CD8 T cells was compared across experiments by normalizing tetramer mean fluorescence intensity (MFI) to the tetramer MFI for CD4+ AV6/BV11 T cells for each experiment. Tetramer MFI was compared to the original $H2^{29.5}$ 2G CAR by two-way ANOVA with Dunnet's multiple comparisons. (**G**) Representative flow staining for CD3γ TRuC CARs. (**H**) % Edited TruC cells after positive selection for NGFR+ cells. (**I**) CD4 and CD8 T cells populations. (**J**) Relative tetramer MFI for TruC T cells compared to CD4+ AV6/BV11 TCR T cells. Data are mean ± SD, (**C-F**) n = 4 independent biological replicates, from 3 independent experiments. (**G-J**) n = 2 replicates from 2 independent experiments.

Supplemental Figure 7.

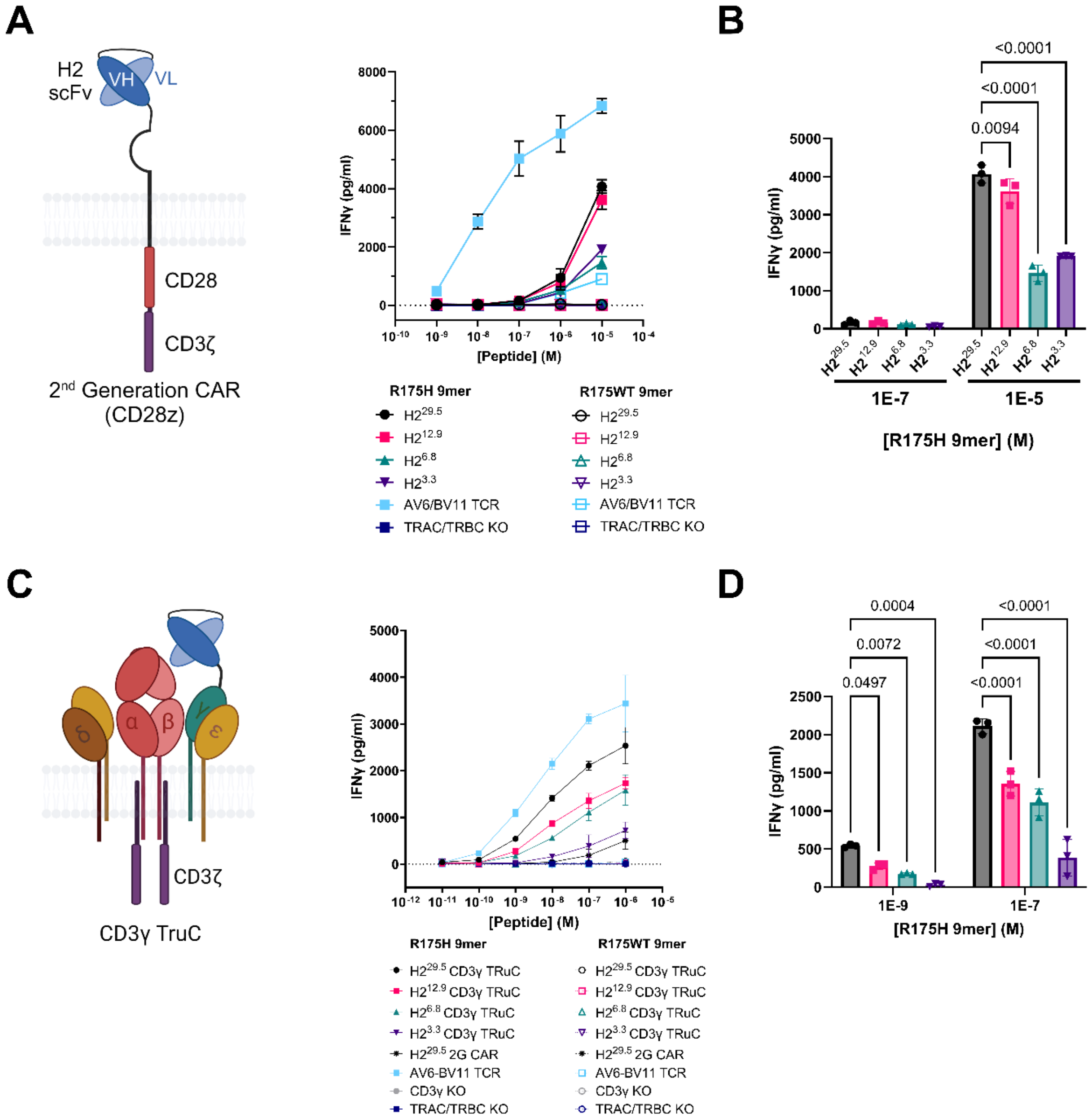

Supplemental Figure 7. Higher affinity 2nd generation CARs and CD3γ-TRuC T cells are less sensitive. (**A**) 8 x 10^3 edited T cells (87.2% editing) were co-cultured with 4 x 10^4 peptide-pulsed T2 target cells for 24 h. Secreted IFN-γ was measured by ELISA. n = 3 per condition, representative of 3 independent experiments. (**B**) IFN-γ values were compared at two peptide pulsing conditions (1E-7 and 1E-5 M p53 R175H 9mer peptide) by two-way ANOVA with Dunnett's multiple comparisons test. n = 3 per condition, representative of 3 independent experiments. (**C**) CD3γ-TRuC T cells were compared to the H2$^{29.5}$ 2nd generation CAR and AV6/BV11 TCR. 1.5 x 10^3 edited T cells (6.7% editing) were co-cultured with 2.5 x 10^3 peptide-pulsed T2 target cells for 24 h. Secreted IFN-γ was measured by ELISA. n = 3 per condition, representative of 2 independent experiments. (**D**) IFN-γ values were compared at two peptide pulsing conditions (1E-9 and 1E-7 M p53 R175H 9mer peptide) by two-way ANOVA with Dunnett's multiple comparisons test. n = 3 per condition, representative of 2 independent experiments.

Supplemental Figure 8.

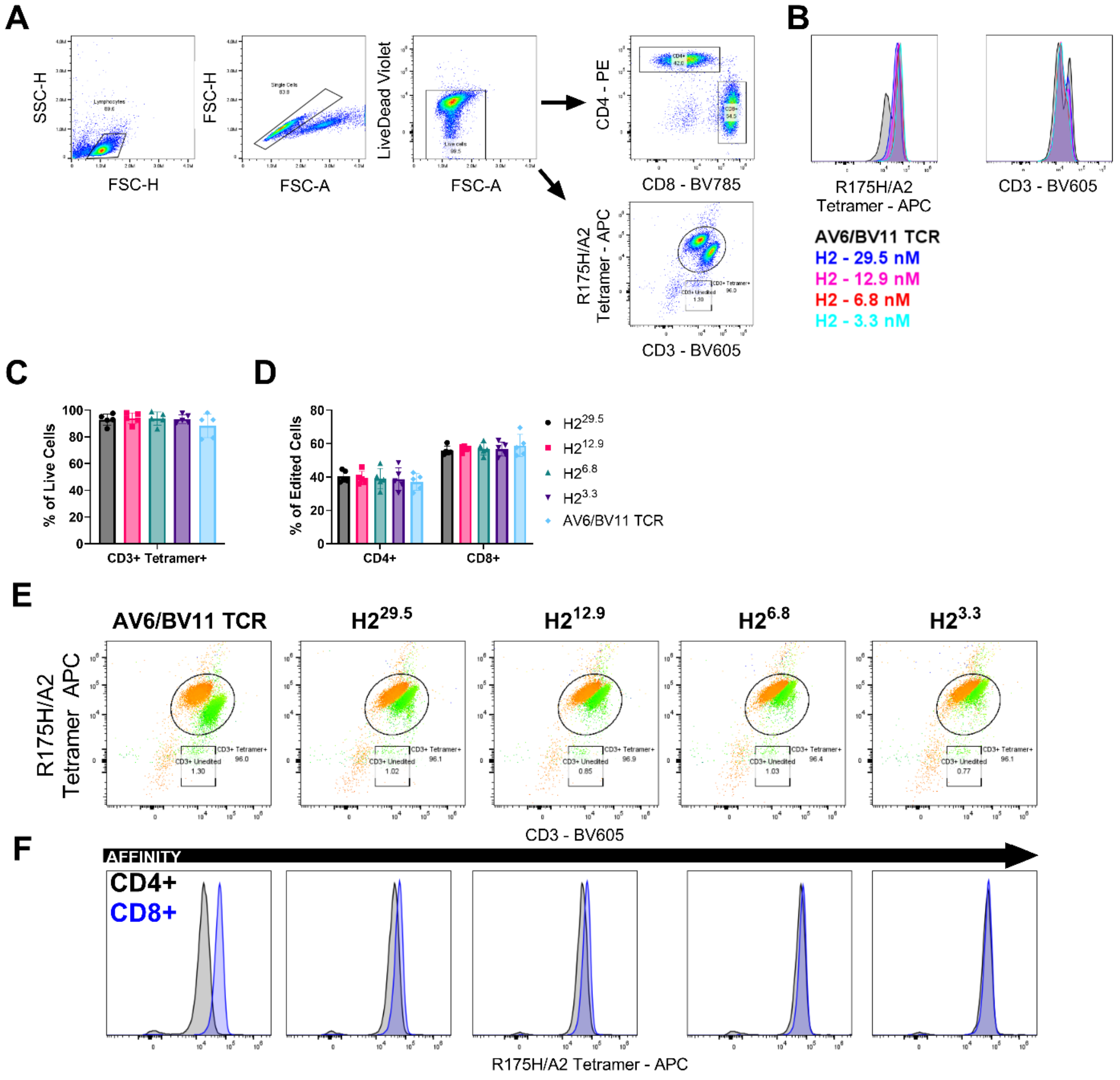

Supplemental Figure 8. STAR binding to R175H/A2 tetramer. (**A**) Gating for flow cytometric analysis of STAR T cells. Edited T cells were purified by anti-NGFR bead selection and characterized by flow cytometry. (**B**) Representative p53 R175H/HLA-A2 tetramer binding and CD3 expression in CD3+ tetramer+ T cells on day 11 post-electroporation. (**C**) Percent edited CD3+ tetramer+ cells after purification is shown for n = 5 independent experiments. (**D**) Percent CD4+ and CD8+ of edited cells, n = 5 independent experiments. (**E**) p53 R175H/HLA-A2 tetramer binding for AV6/BV11 TCR and H2 STAR variants after anti-NGFR-based bead selection (Day 12 post-electroporation). Tetramer binding varies by CD8 expression (CD8 high overlay, orange; CD8 low overlay, green). (**F**) Representative tetramer binding in CD4+ (black) and CD8+ (blue) cells. All data are mean ± SD.

Supplemental Figure 9.

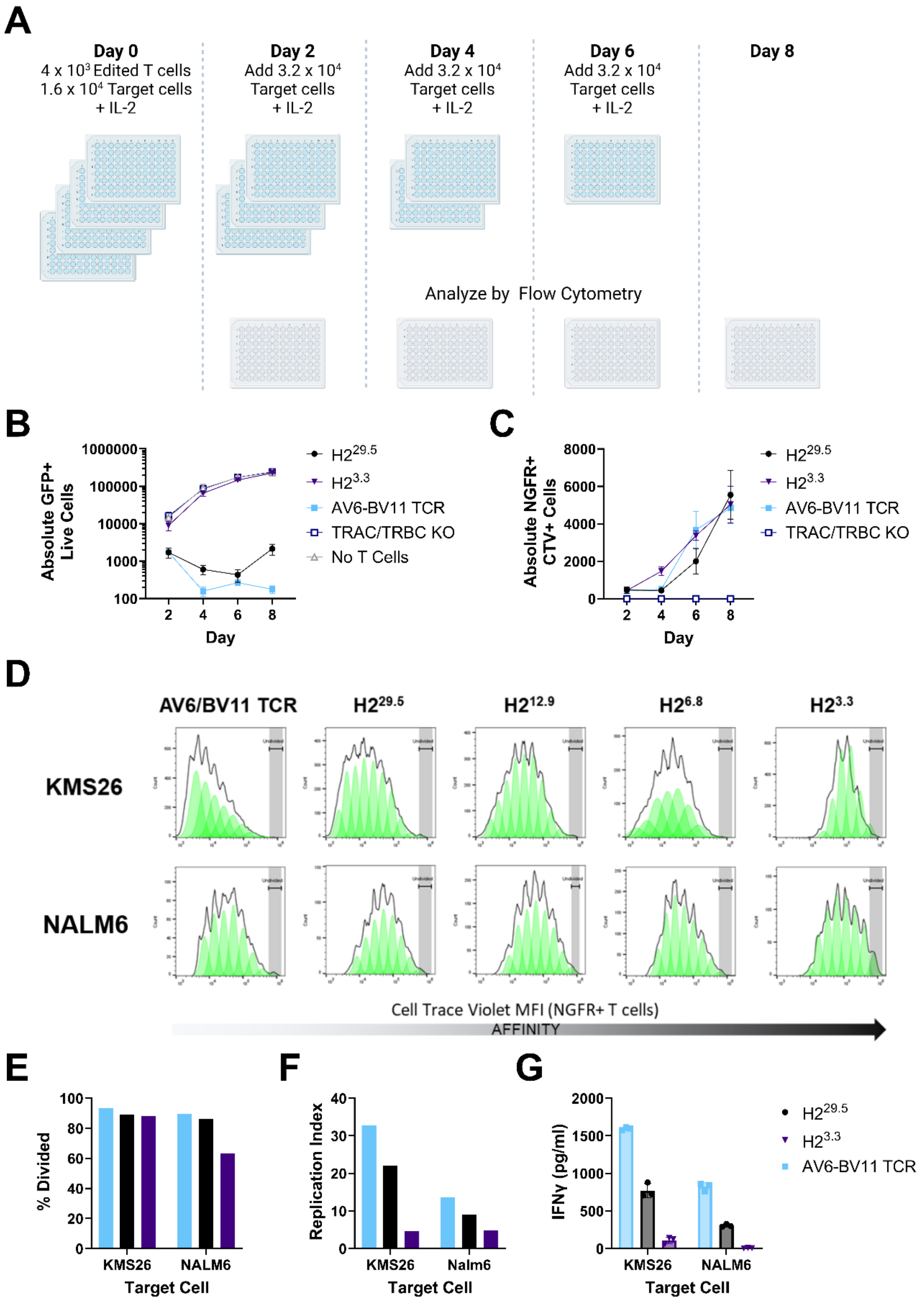

Supplemental Figure 9. Multiple stimulation assay. (**A**) Experimental design. MSA with KMS26 or NALM6 target cells. (**B**) Live GFP+ NALM6^{R175H} target cells were quantified by flow cytometry (n = 3 biological replicates per condition, representative of 3 independent experiments). (**C**) NGFR+ T cells from MSA with NALM6 target cells were quantified by flow cytometry (n = 3 biological replicates, representative of 3 independent experiments). On Day 8 of the MSA, wells were assayed by flow cytometry. (**D**) Replicate wells were concatenated for proliferation analysis in FlowJo v10. (**E**) Percent of NGFR+ T cells that divided and (**F**) replication index are shown for T cells edited with H2$^{29.5}$, H2$^{3.3}$ and AV6/BV11 TCR on Day 8 (n = 3 biological replicates, aggregate data. Representative of 2 independent experiments). (**G**) IFNγ production was quantified by ELISA on Day 2 for KMS26 and NALM6 MSAs (n = 3 biological replicates, single experiment). Data presented are mean ± SD.

Supplemental Figure 10.

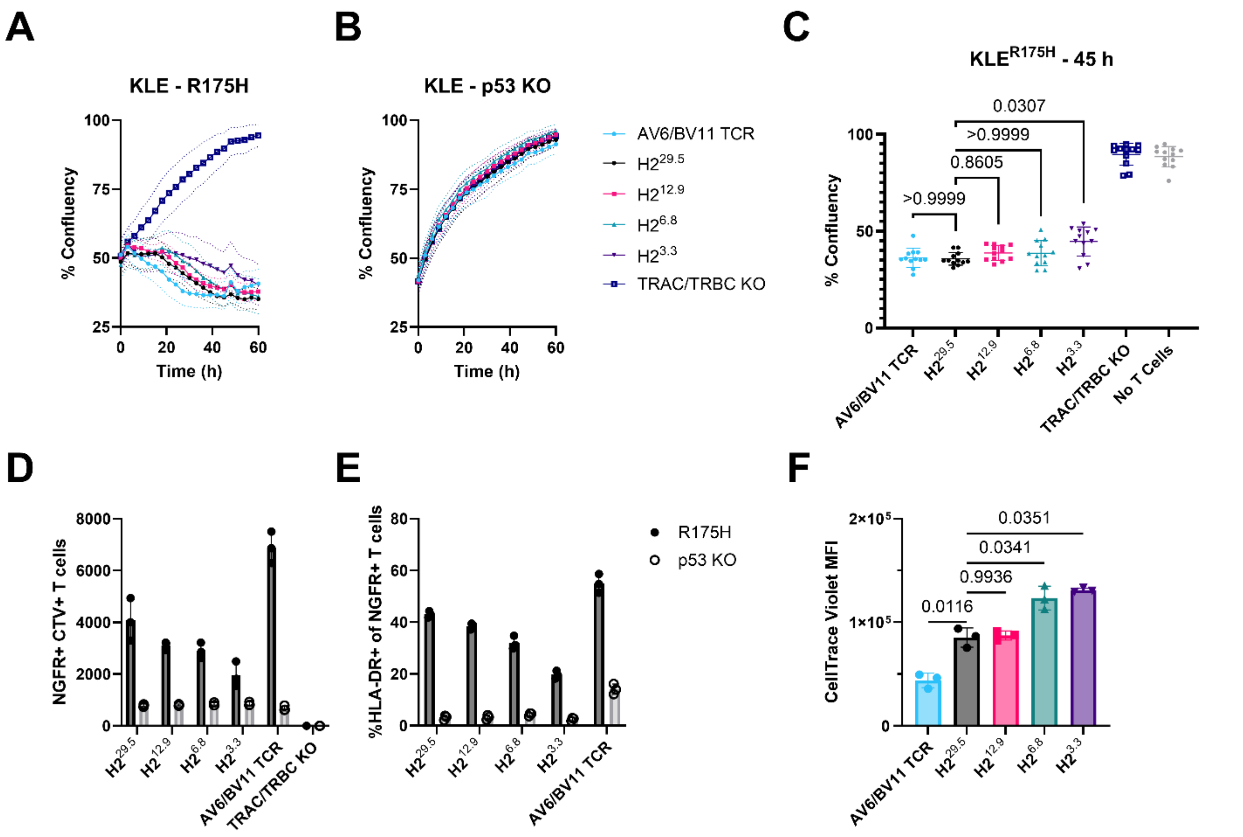

Supplemental Figure 10. STAR T cell killing of KLE target cells. 5 x 10^3 edited T cells were co-cultured with 10^4 KLE target cells for 6 days. Well confluency was measured by high content imaging every 3 h. n = 4 images per well x 3 biological replicate wells, single experiment. Mean ± SD confluency shown through 60 h for (**A**) KLE - R175H and (**B**) KLE - p53 KO. (**C**) Percent confluency at 45 h, mean ranks compared by Kruskal-Wallis test with Dunn's multiple comparisons to H2$^{29.5}$. (**D-F**) Live cells were collected and analyzed by flow cytometry on day 6 (n = 3 biological replicates per well, mean ± SD). (**D**) Count of NGFR+ CellTraceViolet (CTV)+ T cells. (**E**) % of NFGR+ edited T cells expressing HLA-DR. (**F**) CTV MFI of NGFR+ CTV+ HLA-DR+ T cells at day 6 in the KLE - R175H condition. Brown-Forsythe ANOVA with Dunnet's T3 multiple comparisons to H2$^{29.5}$.

Supplemental Figure 11.

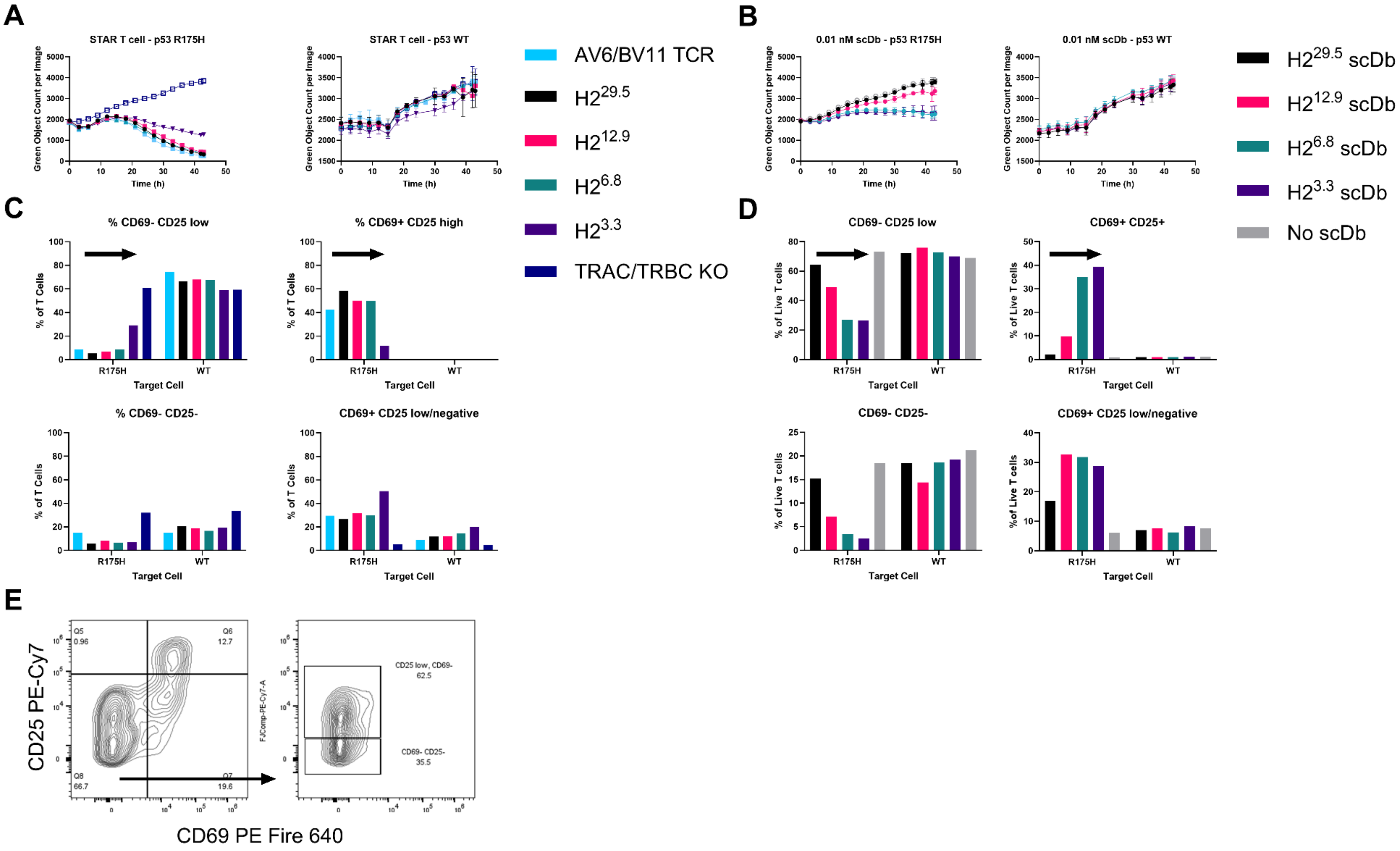

Supplemental Figure 11. CD25 and CD69 upregulation in STAR T cell and scDb co-cultures. 1 x 10^5 GFP+ NALM6 target cells (p53 WT or p53 R175H) were co-cultured with 5 x 10^4 T cells (STAR T cells or unedited T cells plus 0.01 nM scDb) and monitored by high content imaging every 4 h for 2 days. n = 3 biological replicates per condition, representative of 2 independent experiments. After 2 days, cells were assayed for CD69 and CD25 expression by flow cytometry. GFP+ target cells were quantified by high content imaging for (**A**) STAR T cell conditions and (**B**) unedited T cells plus 0.01 nM scDb. (**C-D**) CD25 and CD69 expression [(C) CAR T and (D) scDb, triplicate wells analyzed in aggregate]. (**E**) Flow gating strategy for CD25 and CD69 expression.

Supplemental Figure 12.

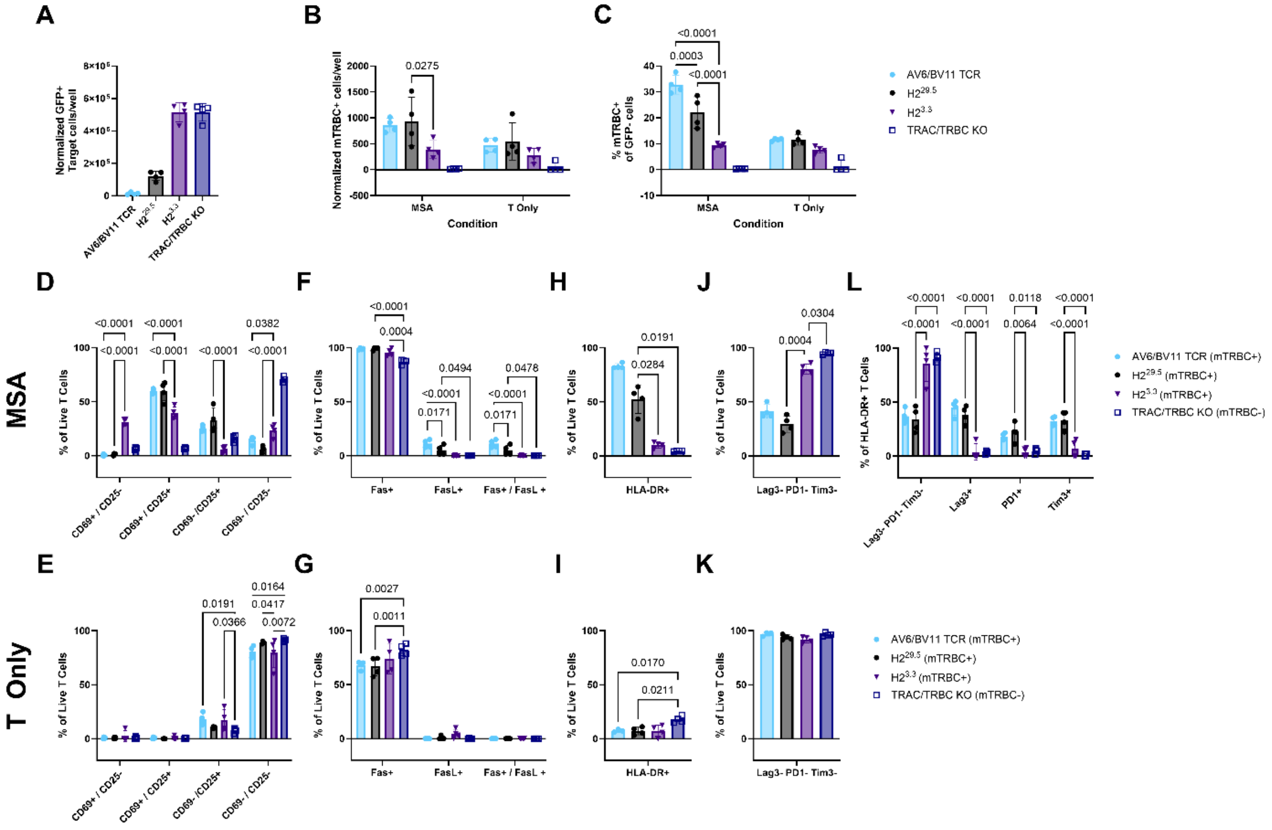

Supplemental Figure 12. Evaluating STAR exhaustion after multiple stimulation assay. 10^4 edited T cells were co-cultured with 5 x 10^4 GFP+ NALM6^{R175H} cells and re-challenged with 5 x 10^4 NALM6^{R175H} cells in the presence of IL-2 every two days until Day 14, when cells were assayed by flow cytometry. Cell counts were normalized to counting beads. (**A**) Live GFP+ target cells on Day 14. (**B**) Count of live STAR T cells expressing murine TRBC (mTRBC) and (**C**) percent mTRBC+ of GFP- T cells. For (**D-K**), AV6/BV11 TCR T cells and STAR T cells were identified as mTRBC+. TRAC/TRBC KO cells were GFP- mTRBC- live cells. (**D-E**) CD69 and CD25 expression in live T cells from MSA (**D**) and T cell only (T only). (**E**) conditions. (**F-G**) Fas (CD95) and FasL (CD95L) expression in T cells from MSA (**F**) and T only (**G**) conditions. (**H-I**) Percent of live T cells expressing HLA-DR from MSA (**H**) and T only (**I**). (**J-K**) Percent of T cells without inhibitory marker expression (Lag- PD1- Tim3-) from MSA (**J**) and T only (**K**). (**L**) Inhibitory marker expression from HLA-DR+ T cells in the MSA condition. Data are mean ± SD with means compared by Two-way ANOVA with Tukey's multiple comparisons. Single experiment, N=4 per condition.

Supplemental Table 1. H2 scFv library variant frequencies determined by Twist Bioscience.

Initial AA			Variant Amino Acid (New Codon) – Variant Frequency by Site																					
Residue	AA	Codon	A (GCT)	C (TGC)	D (GAT)	E (GAG)	F (TTC)	G (GGT)	H (CAC)	I (ATC)	K (AAG)	L (CTG)	M (ATG)	N (AAC)	P (CCT)	Q (CAG)	R (AGA)	S (AGC)	T (ACA)	T (ACC)	V (GTG)	W (TGG)	Y (TAC)	Total
24	R	CGT	0.0400	0.0411	0.0531	0.0568	0.0504	0.0782	0.0404	0.0433	0.0530	0.0411	0.0416	0.0430	0.0492	0.0592	0.0735	0.0456		0.0388	0.0530	0.0533	0.0456	1.0000
25	A	GCA	0.0725	0.0475	0.0519	0.0570	0.0412	0.0569	0.0461	0.0451	0.0396	0.0509	0.0475	0.0346	0.0485	0.0564	0.0490	0.0472		0.0564	0.0507	0.0601	0.0408	1.0000
26	S	AGC	0.0551	0.0611	0.0501	0.0495	0.0527	0.0434	0.0441	0.0695	0.0509	0.0435	0.0503	0.0693	0.0437	0.0455	0.0726	0.0000		0.0537	0.0432	0.0603	0.0417	1.0000
27	Q	CAG	0.0447	0.0347	0.0577	0.0633	0.0471	0.0381	0.0539	0.0432	0.0853	0.0844	0.0567	0.0369	0.0572	0.0000	0.0506	0.0466		0.0347	0.0615	0.0607	0.0428	1.0000
28	D	GAT	0.1207	0.0417	0.0000	0.0761	0.0436	0.0898	0.0346	0.0468	0.0522	0.0465	0.0443	0.0438	0.0481	0.0471	0.0411	0.0487		0.0408	0.0444	0.0501	0.0395	1.0000
29	V	GTT	0.0992	0.0349	0.0771	0.0601	0.0425	0.0793	0.0404	0.0380	0.0421	0.0523	0.0446	0.0336	0.0440	0.0430	0.0482	0.0436		0.0284	0.0584	0.0494	0.0411	1.0000
30	N	AAT	0.0474	0.0239	0.0380	0.0553	0.0517	0.0526	0.0482	0.0495	0.0660	0.0531	0.0499	0.0615	0.0508	0.0599	0.0476	0.0433		0.0461	0.0572	0.0577	0.0403	1.0000
31	T	ACC	0.0529	0.0438	0.0561	0.0454	0.0582	0.0521	0.0467	0.0754	0.0508	0.0361	0.0550	0.0685	0.0259	0.0524	0.0561	0.0559		0.0000	0.0574	0.0682	0.0433	1.0000
32	A	GCA	0.0721	0.0477	0.0664	0.0632	0.0489	0.0591	0.0473	0.0439	0.0568	0.0460		0.0413	0.0517	0.0583	0.0680	0.0453		0.0489	0.0561	0.0358	0.0431	1.0000
33	V	GTT	0.0901	0.0354	0.0759	0.0441	0.0558	0.0791	0.0417	0.0478	0.0428	0.0400	0.0547	0.0390	0.0429	0.0459	0.0476	0.0337		0.0433	0.0619	0.0433	0.0350	1.0000
34	A	GCA	0.0693	0.0373	0.0536	0.0545	0.0414	0.0563	0.0434	0.0445	0.0513	0.0436	0.0482	0.0435	0.0569	0.0536	0.0659	0.0388		0.0463	0.0528	0.0563	0.0426	1.0000
50	S	AGC	0.0510	0.0495	0.0487	0.0577	0.0522	0.0486	0.0348	0.0751	0.0559	0.0540	0.0545	0.0555	0.0450	0.0421	0.0771	0.0000		0.0519	0.0507	0.0533	0.0426	1.0000
51	A	GCC	0.0631	0.0455	0.0523	0.0463	0.0464	0.0545	0.0504	0.0425	0.0449	0.0516	0.0448	0.0480	0.0539	0.0502	0.0437	0.0319		0.0815	0.0556	0.0533	0.0397	1.0000
52	Y	TAC	0.0283	0.1732	0.0492	0.0390	0.1210	0.0398	0.0537	0.0343	0.0423	0.0400	0.0445	0.0522	0.0350	0.0376	0.0479	0.0374		0.0356	0.0418	0.0471	0.0000	1.0000
53	F	TTT	0.0414	0.0380	0.0612	0.0573	0.1020	0.0522	0.0437	0.0503	0.0518	0.0492	0.0472	0.0458	0.0544	0.0391	0.0567	0.0370		0.0363	0.0484	0.0471	0.0408	1.0000
54	L	CTG	0.0530	0.0449	0.0528	0.0550	0.0498	0.0632	0.0446	0.0445	0.0490	0.0000	0.0735	0.0533	0.0515	0.0734	0.0435	0.0441		0.0462	0.0683	0.0524	0.0370	1.0000
55	Y	TAT	0.0457	0.0440	0.0766	0.0502	0.0449	0.0589	0.0408	0.0408	0.0533	0.0561	0.0531	0.0484	0.0490	0.0423	0.0442	0.0457		0.0383	0.0598	0.0490	0.0590	1.0000
56	S	AGC	0.0439	0.0485	0.0534	0.0450	0.0483	0.0653	0.0398	0.0690	0.0517	0.0508	0.0524	0.0624	0.0317	0.0516	0.0846	0.0000		0.0474	0.0454	0.0618	0.0469	1.0000
89	Q	CAG	0.0534	0.0449	0.0514	0.0585	0.0376	0.0600	0.0619	0.0439	0.0792	0.0697	0.0478	0.0420	0.0475	0.0000	0.0410	0.0464		0.0374	0.0550	0.0762	0.0461	1.0000
90	Q	CAA	0.0544	0.0423	0.0623	0.0516	0.0480	0.0564	0.0701	0.0454	0.0473	0.0495	0.0453	0.0423	0.0476	0.0603	0.0528	0.0438		0.0351	0.0554	0.0436	0.0465	1.0000
91	Y	TAC	0.0448	0.0966	0.0555	0.0524	0.0823	0.0551	0.0555	0.0452	0.0587	0.0397	0.0424	0.0545	0.0483	0.0424	0.0454	0.0410		0.0352	0.0498	0.0552	0.0000	1.0000
92	S	TCT	0.1033	0.0409	0.0530	0.0433	0.0528	0.0548	0.0383	0.0373	0.0373	0.0472	0.0361	0.0399	0.0850	0.0401	0.0450	0.0465		0.0490	0.0513	0.0530	0.0458	1.0000
93	R	AGA	0.0542	0.0491	0.0541	0.0561	0.0426	0.0702	0.0385	0.0526	0.0513	0.0447	0.0503	0.0447	0.0570	0.0544	0.0000	0.0722		0.0511	0.0562	0.0601	0.0406	1.0000
94	Y	TAC	0.0484	0.1003	0.0552	0.0552	0.0830	0.0517	0.0460	0.0420	0.0548	0.0407	0.0366	0.0603	0.0463	0.0442	0.0508	0.0445		0.0419	0.0488	0.0496	0.0000	1.0000
95	S	TCT	0.0965	0.0401	0.0555	0.0537	0.0446	0.0591	0.0348	0.0402	0.0524	0.0471	0.0459	0.0419	0.0710	0.0477	0.0529	0.0433		0.0425	0.0501	0.0384	0.0423	1.0000
96	P	CCT	0.0609	0.0418	0.0606	0.0539	0.0462	0.0628	0.0539	0.0482	0.0522	0.0576	0.0527	0.0430	0.0000	0.0647	0.0415	0.0434		0.0503	0.0557	0.0577	0.0527	1.0000
97	V	GTT	0.0726	0.0449	0.0820	0.0601	0.0436	0.0676	0.0402	0.0440	0.0411	0.0463	0.0495	0.0393	0.0422	0.0417	0.0463	0.0459		0.0388	0.0662	0.0529	0.0347	1.0000
151	F	TTT	0.0483	0.0391	0.0536	0.0519	0.0616	0.0623	0.0458	0.0381	0.0476	0.0570	0.0482	0.0443	0.0481	0.0540	0.0515	0.0436		0.0432	0.0601	0.0610	0.0407	1.0000
152	N	AAC	0.0405	0.0394	0.0451	0.0484	0.0402	0.0505	0.0583	0.0598	0.0667	0.0386	0.0465	0.0000	0.0479	0.0451	0.0508	0.0718		0.0742	0.0484	0.0518	0.0762	1.0000
153	V	GTT	0.0783	0.0404	0.0684	0.0427	0.0464	0.1207	0.0327	0.0405	0.0422	0.0488	0.0414	0.0411	0.0420	0.0409	0.0382	0.0386		0.0418	0.0672	0.0489	0.0388	1.0000
154	Y	TAC	0.0053	0.0851	0.0535	0.0614	0.0924	0.0431	0.0546	0.0544	0.0508	0.0543	0.0464	0.0577	0.0463	0.0524	0.0512	0.0479		0.0413	0.0483	0.0535	0.0000	1.0000
155	A	GCA	0.0637	0.0409	0.0561	0.0493	0.0471	0.0620	0.0416	0.0435	0.0478	0.0530	0.0396	0.0420	0.0558	0.0515	0.0626	0.0495		0.0482	0.0584	0.0420	0.0455	1.0000
156	S	TCT	0.0843	0.0432	0.0563	0.0514	0.0516	0.0544	0.0418	0.0417	0.0513	0.0440	0.0459	0.0396	0.0668	0.0476	0.0493	0.0419		0.0411	0.0474	0.0520	0.0483	1.0000
157	G	GGT	0.0543	0.0583	0.0843	0.0512	0.0449	0.0000	0.0464	0.0415	0.0431	0.0502	0.0431	0.0466	0.0526	0.0499	0.0545	0.0600		0.0408	0.0589	0.0664	0.0530	1.0000
158	M	ATG	0.0405	0.0481	0.0453	0.0510	0.0461	0.0656	0.0396	0.0500	0.0648	0.0793	0.0000	0.0470	0.0476	0.0512	0.0575	0.0510		0.0367	0.0802	0.0636	0.0348	1.0000
159	H	CAT	0.0568	0.0347	0.0727	0.0473	0.0409	0.0543	0.0630	0.0457	0.0453	0.0508	0.0514	0.0417	0.0811	0.0711	0.0391	0.0374		0.0394	0.0436	0.0411	0.0425	1.0000
172	V	GTT	0.0931	0.0331	0.0739	0.0530	0.0468	0.0846	0.0392	0.0383	0.0398	0.0391	0.0517	0.0395	0.0437	0.0458	0.0438	0.0409		0.0475	0.0599	0.0479	0.0385	1.0000
173	A	GCC	0.0776	0.0424	0.0432	0.0481	0.0553	0.0457	0.0483	0.0390	0.0416	0.0480	0.0467	0.0394	0.0433	0.0479	0.0409	0.0462		0.0903	0.0532	0.0555	0.0475	1.0000
174	K	AAA	0.0451	0.0399	0.0520	0.0454	0.0417	0.0450	0.0451	0.0490	0.0670	0.0464	0.0432	0.0630	0.0353	0.0472	0.1263	0.0389		0.0345	0.0529	0.0347	0.0474	1.0000
175	I	ATC	0.0420	0.0432	0.0524	0.0538	0.0715	0.0447	0.0389	0.0000	0.0542	0.0492	0.0456	0.0700	0.0479	0.0532	0.0488	0.0783		0.0581	0.0585	0.0477	0.0418	1.0000
176	Y	TAC	0.0457	0.1404	0.0429	0.0477	0.1064	0.0481	0.0599	0.0342	0.0427	0.0384	0.0435	0.0425	0.0342	0.0486	0.0417	0.0483		0.0358	0.0473	0.0516	0.0000	1.0000
177	P	CCA	0.0585	0.0451	0.0485	0.0459	0.0530	0.0645	0.0490	0.0459	0.0476	0.0490	0.0458	0.0516	0.0693	0.0459	0.0562	0.0444		0.0400	0.0480	0.0495	0.0420	1.0000
178	D	GAT	0.0831	0.0488	0.0000	0.0846	0.0391	0.0829	0.0385	0.0482	0.0596	0.0515	0.0467	0.0525	0.0529	0.0543	0.0582	0.0471		0.0454	0.0634		0.0432	1.0000
179	S	TCT	0.0752	0.0432	0.0521	0.0504	0.0497	0.0489	0.0439	0.0401	0.0487	0.0459	0.0493	0.0408	0.0698	0.0534	0.0458	0.0483		0.0427	0.0457	0.0557	0.0504	1.0000
180	D	GAT	0.0797	0.0403	0.0000	0.0757	0.0418	0.0870	0.0508	0.0440	0.0408	0.0518	0.0545	0.0412	0.0456	0.0554	0.0500	0.0544		0.0386	0.0555	0.0504	0.0425	1.0000
181	Y	TAC	0.0446	0.0759	0.0517	0.0480	0.0754	0.0643	0.0605	0.0419	0.0498	0.0389	0.0457	0.0535	0.0474	0.0532	0.0495	0.0503		0.0407	0.0546	0.0543	0.0000	1.0000
182	T	ACC	0.0623	0.0493	0.0546	0.0540	0.0486	0.0616	0.0448	0.0551	0.0504	0.0457	0.0418	0.0585	0.0552	0.0551	0.0543	0.0470		0.0000	0.0592	0.0617	0.0408	1.0000
183	Y	TAC	0.0447	0.1050	0.0509	0.0594	0.0829	0.0477	0.0625	0.0484	0.0451	0.0396	0.0380	0.0621	0.0455	0.0472	0.0387	0.0415		0.0452	0.0442	0.0515	0.0000	1.0000
184	Y	TAT	0.0511	0.0328	0.0845	0.0565	0.0483	0.0442	0.0233	0.0398	0.0676	0.0448	0.0562	0.0403	0.0505	0.0526	0.0479	0.0429		0.0291	0.0567	0.0580	0.0731	1.0000
221	S	AGC	0.0489	0.0595	0.0525	0.0499	0.0447	0.0570	0.0471	0.0646	0.0540	0.0467	0.0516	0.0567	0.0409	0.0409	0.0812	0.0000		0.0502	0.0528	0.0583	0.0425	1.0000
222	R	CGC	0.0452	0.0767	0.0445	0.0546	0.0410	0.0529	0.0699	0.0462	0.0393	0.0538	0.0454	0.0352	0.0353	0.0494	0.0562	0.0710		0.0377	0.0487	0.0588	0.0382	1.0000
223	D	GAT	0.0788	0.0370	0.0000	0.0728	0.0444	0.0753	0.0484	0.0429	0.0455	0.0492	0.0471	0.0512	0.0515	0.0472	0.0601	0.0442		0.0467	0.0606	0.0556	0.0415	1.0000
224	S	TCT	0.0658	0.0402	0.0590	0.0483	0.0473	0.0575	0.0430	0.0446	0.0492	0.0513	0.0596	0.0397	0.0547	0.0526	0.0510	0.0345		0.0475	0.0555	0.0579	0.0409	1.0000
225	S	TCT	0.0803	0.0491	0.0537	0.0400	0.0399	0.0548	0.0373	0.0386	0.0586	0.0438	0.0469	0.0462	0.0681	0.0459	0.0564	0.0430		0.0386	0.0566	0.0618	0.0404	1.0000
226	F	TTT	0.0412	0.0417	0.0576	0.0469	0.0636	0.0571	0.0491	0.0450	0.0498	0.0548	0.0495	0.0445	0.0412	0.0543	0.0490	0.0471		0.0488	0.0583	0.0588	0.0419	1.0000
227	Y	TAC	0.0448	0.1051	0.0367	0.0533	0.1068	0.0516	0.0624	0.0389	0.0417	0.0412	0.0494	0.0575	0.0425	0.0473	0.0448	0.0427		0.0374	0.0444	0.0514	0.0000	1.0000
228	Y	TAC	0.0471	0.0829	0.0448	0.0542	0.0772	0.0552	0.0577	0.0400	0.0487	0.0574	0.0454	0.0511	0.0381	0.0566	0.0387	0.0473		0.0503	0.0542	0.0532	0.0000	1.0000
229	V	GTT	0.0702	0.0395	0.0607	0.0461	0.0507	0.0807	0.0462	0.0478	0.0403	0.0544	0.0489	0.0348	0.0487	0.0460	0.0466	0.0415		0.0392	0.0719	0.0457	0.0399	1.0000
230	Y	TAC	0.0502	0.0796	0.0496	0.0542	0.0661	0.0612	0.0562	0.0342	0.0425	0.0483	0.0525	0.0531	0.0524	0.0545	0.0525	0.0473		0.0439	0.0494	0.0525	0.0000	1.0000
231	A	GCA	0.0596	0.0405	0.0555	0.0590	0.0493	0.0664	0.0406	0.0412	0.0463	0.0492	0.0442	0.0422	0.0448	0.0390	0.0505	0.0445	0.0765		0.0635	0.0449	0.0422	1.0000
232	M	ATG	0.0556	0.0398	0.0477	0.0536	0.0514	0.0486	0.0449	0.0524	0.0665	0.0699	0.0000	0.0465	0.0462	0.0510	0.0571	0.0511		0.0496	0.0706	0.0585	0.0390	1.0000

Supplemental Table 2. R175H peptide EC50s for H2 variant bispecific antibodies

Variant scDb	EC50 ([R175H 9mer], M)	R squared
Original H2	1.606E-09	0.9756
R24D	5.319E-11	0.9581
S26P	8.445E-11	0.8878
V29D	3.893E-11	0.9519
V29E	1.149E-11	0.9263
F53S	2.464E-10	0.9912
Q90D	1.987E-12	0.9241
Y57I	1.586E-10	0.8693
Y57L	1.665E-10	0.8445

Supplemental Table 3. Plasmid sequences (see separate excel file).

Supplemental Table 4. CRISPR Guide RNAs

Name	Description	Sequence
Cpf1 *TRAC* gRNA	Cpf1 A.s. crRNA	GAGTCTCTCAGCTGGTACAC
Cpf1 *TRBC* gRNA	Cpf1 A.s. crRNA	GCCCTATCCTGGGTCCACTC
Cpf1 *CD3G* gRNA	Cpf1 A.s. crRNA	CAGGTACTTTGGCCCAGTCA
